# A versatile drug delivery system targeting senescent cells

**DOI:** 10.15252/emmm.201809355

**Published:** 2018-07-16

**Authors:** Daniel Muñoz‐Espín, Miguel Rovira, Irene Galiana, Cristina Giménez, Beatriz Lozano‐Torres, Marta Paez‐Ribes, Susana Llanos, Selim Chaib, Maribel Muñoz‐Martín, Alvaro C Ucero, Guillermo Garaulet, Francisca Mulero, Stephen G Dann, Todd VanArsdale, David J Shields, Andrea Bernardos, José Ramón Murguía, Ramón Martínez‐Máñez, Manuel Serrano

**Affiliations:** ^1^ Tumor Suppression Group Spanish National Cancer Research Centre (CNIO) Madrid Spain; ^2^ CRUK Cambridge Centre Early Detection Programme Department of Oncology Hutchison/MRC Research Centre University of Cambridge Cambridge UK; ^3^ Cellular Plasticity and Disease Group Institute for Research in Biomedicine (IRB Barcelona) Barcelona Institute of Science and Technology (BIST) Barcelona Spain; ^4^ Instituto Interuniversitario de Investigación de Reconocimiento Molecular y Desarrollo Tecnológico (IDM) Universitat Politècnica de València Universitat de València Valencia Spain; ^5^ CIBER de Bioingeniería, Biomateriales y Nanomedicina (CIBER‐BBN) Spain; ^6^ Genes, Development and Disease Group Spanish National Cancer Research Centre (CNIO) Madrid Spain; ^7^ Molecular Imaging Unit Spanish National Cancer Research Centre (CNIO) Madrid Spain; ^8^ Oncology R&D Group, Pfizer Worldwide Research & Development Pfizer Inc. La Jolla CA USA; ^9^ Departamento de Química Universitat Politècnica de València Valencia Spain; ^10^ Catalan Institution for Research and Advanced Studies (ICREA) Barcelona Spain

**Keywords:** chemotherapy, fibrosis, nanomedicine, palbociclib, senescence, Ageing, Pharmacology & Drug Discovery

## Abstract

Senescent cells accumulate in multiple aging‐associated diseases, and eliminating these cells has recently emerged as a promising therapeutic approach. Here, we take advantage of the high lysosomal β‐galactosidase activity of senescent cells to design a drug delivery system based on the encapsulation of drugs with galacto‐oligosaccharides. We show that gal‐encapsulated fluorophores are preferentially released within senescent cells in mice. In a model of chemotherapy‐induced senescence, gal‐encapsulated cytotoxic drugs target senescent tumor cells and improve tumor xenograft regression in combination with palbociclib. Moreover, in a model of pulmonary fibrosis in mice, gal‐encapsulated cytotoxics target senescent cells, reducing collagen deposition and restoring pulmonary function. Finally, gal‐encapsulation reduces the toxic side effects of the cytotoxic drugs. Drug delivery into senescent cells opens new diagnostic and therapeutic applications for senescence‐associated disorders.

## Introduction

Severe or unrepairable cellular damage often triggers a stereotypic cellular response known as senescence. This cellular program is controlled by relatively well‐understood pathways that stably block cell proliferation and are conserved across vertebrates (Muñoz‐Espín & Serrano, 2014). The main purpose of cellular senescence is to prevent the proliferation of damaged cells and, at the same time, to trigger tissue repair through the secretion of a complex mixture of extracellular factors, known as senescence‐associated secretory phenotype or SASP. Specifically, senescence‐initiated tissue repair involves the recruitment of inflammatory cells, the dismissal of senescent cells by phagocytic and immune cells, and the activation of stem/progenitor features in non‐damaged surrounding cells (Krizhanovsky *et al*, 2008; Demaria *et al*, 2014; Yun *et al*, 2015; Mosteiro *et al*, 2016; Chiche *et al*, 2017; Ritschka *et al*, 2017). Even during vertebrate development, embryos use senescence in a damage‐independent manner to initiate specific tissue remodeling processes (Muñoz‐Espín *et al*, 2013; Storer *et al*, 2013).

Upon persistent damage or during aging, senescent cells accumulate, probably due to an inefficient clearance by immune cells, and this accumulation may lead to chronic inflammation and fibrosis (Muñoz‐Espín & Serrano, 2014). Indeed, evidence in mice indicates that the accumulation of senescent cells actively contributes to multiple diseases and aging (Muñoz‐Espín & Serrano, 2014). In this regard, genetic ablation of senescent cells delays and ameliorates some aging‐associated diseases, reverts long‐term degenerative processes associated with chemotherapy, and extends longevity (Baker *et al*, 2016; Childs *et al*, 2016; Demaria *et al*, 2017). Importantly, senescent cells present vulnerabilities to particular small molecule inhibitors, known as “senolytics”, that trigger apoptosis preferentially in senescent cells (Zhu *et al*, 2015). For example, the combination of dasatinib (a tyrosine kinase inhibitor of broad specificity) and quercetin (a flavonol with antioxidant and estrogenic activity) has preferential toxicity over senescent cells compared to control cells (Zhu *et al*, 2015). Also, the survival of senescent cells is highly dependent on elevated levels of the BCL‐2 family of anti‐apoptotic factors, and accordingly, senescent cells are hyper‐sensitive to apoptosis induced by navitoclax, a BCL‐2 family inhibitor (Chang *et al*, 2016; Yosef *et al*, 2016; Zhu *et al*, 2016; Pan *et al*, 2017). Similarly, inactivation of the transcription factor FOXA4 with a peptide derivative preferentially eliminates senescent cells over non‐senescent ones (Baar *et al*, 2017). These pharmacological treatments reduce the number of senescent cells *in vivo* and show therapeutic activity against senescence‐associated diseases and aging (Zhu *et al*, 2015, 2016; Chang *et al*, 2016; Roos *et al*, 2016; Yosef *et al*, 2016; Baar *et al*, 2017; Pan *et al*, 2017; Schafer *et al*, 2017).

Senescent cells *in vitro* are characterized by high levels of lysosomal β‐galactosidase activity, known as senescence‐associated β‐galactosidase (SAβGal; Dimri *et al*, 1995; Muñoz‐Espín & Serrano, 2014; Kurz *et al*, 2000; Lee *et al*, 2006). In addition to β‐galactosidase, senescent cells present high levels of most tested lysosomal hydrolases (Knaś *et al*, 2012). Indeed, senescent cells show a remarkable accumulation of lysosomes, together with abnormal endosomal traffic and autophagy (Cho *et al*, 2011; Narita *et al*, 2011; Ivanov *et al*, 2013; Udono *et al*, 2015; Tai *et al*, 2017). Interestingly, damaged or diseased tissues generally contain cells that are positive for SAβGal, while normal healthy tissues are negative for this marker (Sharpless & Sherr, 2015).

Here, we have explored the possibility of using lysosomal β‐galactosidase as a vulnerable trait of senescent cells that can be exploited to deliver tracers or drugs preferentially to diseased tissues with high content of senescent cells. Our approach is based on the encapsulation of diagnostic or therapeutic agents with β(1,4)‐galacto‐oligosaccharides and their delivery to lysosomes via endocytosis. We show that this delivery strategy is effective *in vivo*, and it is therapeutic in the context of cancer chemotherapy and also against pulmonary fibrosis. Moreover, our encapsulation system has the added value of reducing the systemic toxicities of cytotoxic drugs.

## Results

### Validation of gal‐encapsulation to target senescent cells *in vitro*

**Figure 1:**
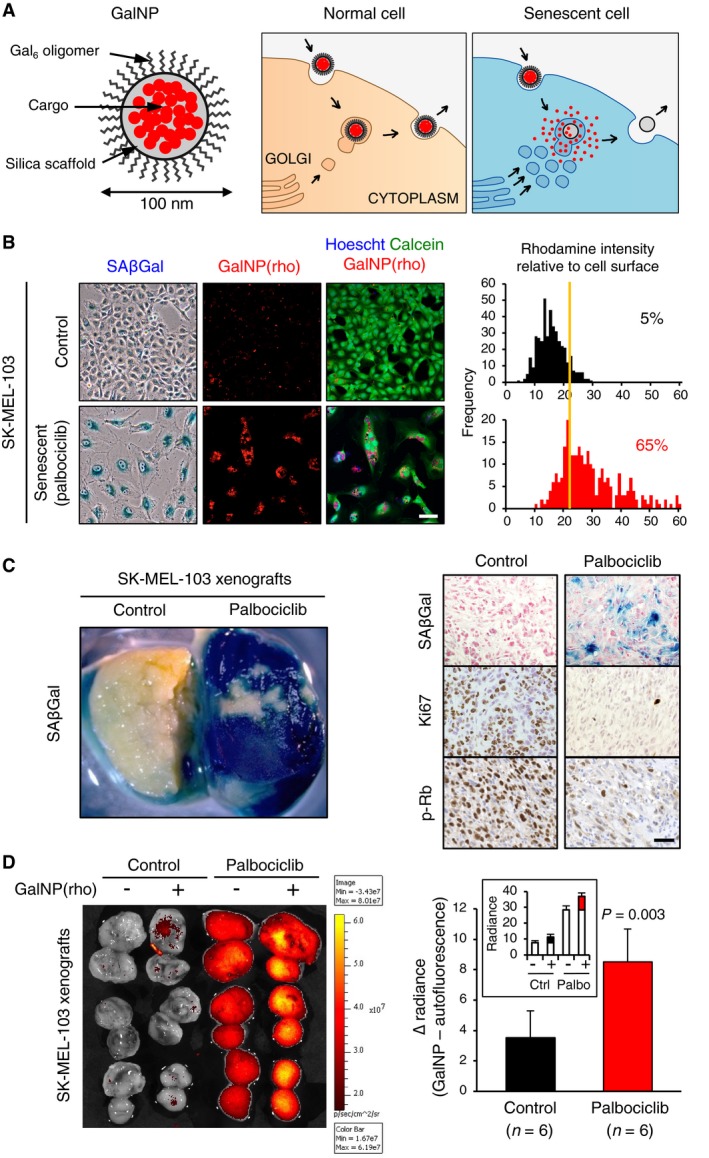
Release of gal‐encapsulated fluorophores in xenografts AGalNP beads are based on a mesoporous silica scaffold (MCM‐41) that can be loaded with different cargoes encapsulated by a coat of 6‐mer β(1,4)‐galacto‐oligosaccharides. Cellular uptake of the GalNP beads occurs via endocytosis and, after fusion with lysosomal vesicles, the beads are released by exocytosis. The high lysosomal β‐galactosidase activity of senescent cells allows a preferential release of the cargo by a β‐galactosidase‐mediated hydrolysis of the cap.BSK‐MEL‐103 melanoma cells were treated with palbociclib (1 μM) for 1 week, and senescence induction was assessed by SAβgal staining. Next, cultures were exposed to GalNP(rho) (50 μg/ml, for 16 h). Pictures show representative images illustrating rhodamine release by confocal microscopy. Cells were co‐stained with Calcein, and nuclei were stained with Hoechst. Graphs to the right show the rhodamine intensity relative to cell surface in senescent cells and non‐senescent (control) cells. Each assay was repeated at least three times with similar results. Scale bar: 50 μm.CSubcutaneous tumor xenografts of SK‐MEL‐103 melanoma cells in athymic female nude mice. Upon tumor formation, mice were treated daily with palbociclib (oral gavage, 100 mg/kg) during 7 days. The left panel picture shows representative whole tissue portions of tumors after SAβGal staining. The right panel shows sections of control and palbociclib‐treated tumors processed for SAβGal staining, and Ki67 and phosphorylated Rb (p‐Rb) immunohistochemistry. This experiment has been repeated at least two times with similar results. Scale bar: 50 μm.DMice bearing SK‐MEL‐103 xenografts, control or treated with palbociclib for 7 days, as in (C), were tail vein injected with 200 μl of a solution containing GalNP(rho) (4 mg/ml). At 6 h post‐injection, mice were sacrificed, tumors were collected, and fluorescence was analyzed by an IVIS spectrum imaging system. The graph indicates the average difference in tumor radiance between GalNP‐injected control and palbociclib‐treated groups. The inset shows the absolute values of radiance (p/s/cm^2^/sr × 10^6^) for each group. The corresponding differences are highlighted in black or red. Values are expressed as mean ± SD, and statistical significance was assessed by the two‐tailed Student's *t*‐test.

Sugar‐coated beads (~100 nm diameter), based on a silica porous scaffold known as MCM‐41 (Kresge *et al*, 1992), are efficiently internalized into cells by endocytosis, targeted to the lysosomes, and eventually released by exocytosis (Slowing *et al*, 2006, 2011; Tao *et al*, 2009; Bernardos *et al*, 2010; Hocine *et al*, 2010; Yanes *et al*, 2013; Aznar *et al*, 2016). In a previous work (Agostini *et al*, 2012), we designed beads pre‐loaded with a fluorophore and then coated with a layer of galacto‐oligosaccharides of mixed lengths (referred to as GosNP). We observed that β‐galactosidase can digest the sugar coating of the beads, thus allowing the diffusion of the cargo out of the silica scaffold. Interestingly, fluorophore‐loaded GosNP efficiently release their content within senescent cells, in agreement with the high levels of β‐galactosidase activity of these cells (Agostini *et al*, 2012). Here, we have improved this system by using a homogeneous coating mostly consisting of a 6‐mer galacto‐oligosaccharide (referred to as GalNP) (Fig 1A and Appendix Fig S1A). Using this strategy, we have encapsulated rhodamine B [GalNP(rho)], doxorubicin [GalNP(dox)], and navitoclax [GalNP(nav)], and enzymatic digestion of the three types of beads with fungal β‐galactosidase demonstrated efficient release of their respective cargos (Appendix Fig S1B–E). In general, 100 mg of drug is encapsulated per gram of beads and, upon digestion *in vitro*, ~30 mg of drug is released per gram of beads (Appendix Tables S1–S3). Targeting of senescent cells with GalNP(rho) was validated in three human cancer cell lines treated with palbociclib (a selective CDK4/CDK6 inhibitor approved in combination with letrozole or fulvestrant for hormone receptor^+^/HER2^−^ metastatic breast cancer; Fig 1B and Appendix Fig S1F and G). Of note, the three cell lines used, SK‐MEL‐103, NCI‐H226, and UT‐SCC‐42B, have an active retinoblastoma pathway (RB1‐proficient; Ikediobi *et al*, 2006; Giefing *et al*, 2011; Massaro *et al*, 2017) and undergo senescence upon treatment with palbociclib (Fig 1B and Appendix Fig S1F). In these cellular models, the fluorophore was released more efficiently in senescent cells compared to control cells, demonstrating the functionality of the GalNP encapsulation method. Co‐staining of cells with a lysosomal marker indicated that a substantial fraction of rhodamine was present in lysosomes (Appendix Fig S1H).

### Release of gal‐encapsulated fluorophores in xenografts

**Figure 2:**
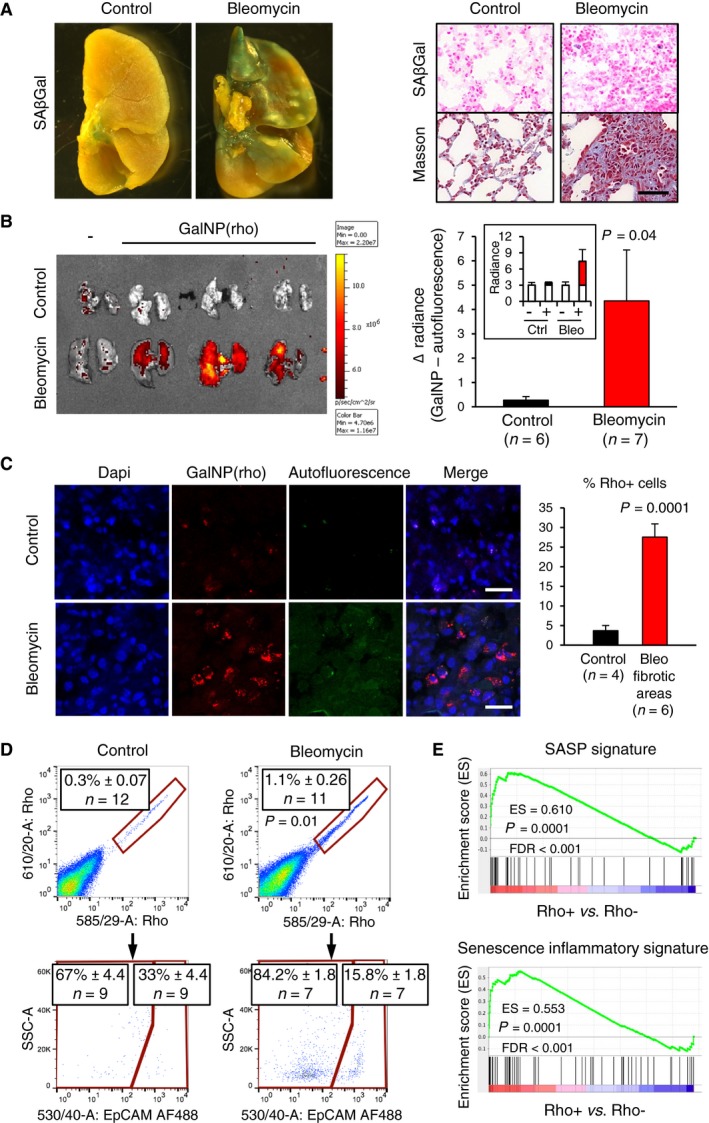
Release of gal‐encapsulated fluorophores in fibrotic lungs AC57BL/6 male mice were subjected to a single intratracheal administration bleomycin (1.5 U/kg) and analyzed 2 weeks later. Pictures at the left correspond to representative lungs after whole tissue SAβGal staining. The right panel shows sections of control and bleomycin‐treated lungs processed for SAβGal and Masson's trichrome staining to detect collagen fibers (stained in blue). Scale bar: 50 μm.BControl and bleomycin‐treated mice, as in (A), were tail vein injected with 200 μl of a solution containing GalNP(rho) (4 mg/ml). At 6 h post‐injection, mice were sacrificed and the lungs were analyzed by an IVIS spectrum imaging system, as in Fig 1D. The graph indicates the average difference in lung radiance between GalNP‐injected control and bleomycin‐treated groups. The inset shows the absolute values of radiance (p/s/cm^2^/sr × 10^6^) for each group. The corresponding differences are highlighted in black or red. Values are expressed as mean ± SD, and statistical significance was assessed by the two‐tailed Student's *t*‐test.CControl and bleomycin‐treated mice were injected with GalNP(rho), as in (B), and lung sections were analyzed 6 h post‐injection by confocal microscopy. Pictures correspond to representative sections of control and bleomycin‐treated lungs. The graph shows % of rhodamine^+^ cells in fibrotic areas from bleomycin‐treated mice compared to normal lung tissue from control mice. Values are expressed as mean ± SD, and statistical significance was assessed by the two‐tailed Student's *t*‐test. Scale bar: 25 μm.DLung cell suspensions from control and bleomycin‐treated mice, as in (B), were analyzed by flow cytometry. The upper panels show representative dot plots of rhodamine staining in CD45^−^CD31^−^ cells. The gating strategy is shown in detail in Appendix Fig S2C using as example the panel corresponding to the bleomycin‐treated lung shown here. Values in boxes correspond to the mean ± SEM, and statistical significance was assessed by the two‐tailed Student's *t*‐test. The lower panels show representative dot plots of rhodamine^+^ cells (after exclusion of CD45^+^ and CD31^+^ cells) separated in EpCAM^−^ (fibroblasts) and EpCAM^+^ (epithelial cells) subpopulations. Values in boxes correspond to the mean ± SEM of these two populations. Statistical significance between the fibroblast:epithelial ratio of positivity was assessed by the two‐sided Fisher exact test (*P* = 0.008).EGSEA plots of published signatures of SASP and SIR (senescence‐inflammatory response) (Lasry & Ben‐Neriah, 2015) against the ranked list of differential expression between Rho^+^ and Rho^−^ cells (all CD45^−^CD31^−^) from bleomycin‐treated mice (*n* = 3), at 2 weeks post‐bleomycin, as in (D).

To evaluate the release of gal‐encapsulated fluorophores *in vivo*, we employed tumor xenografts treated with senescence‐inducing chemotherapy. Subcutaneous xenografts were generated using SK‐MEL‐103 melanoma cells and NCI‐H226 lung squamous carcinoma cells. Upon tumor formation, mice were treated daily with palbociclib for 7 days, and this resulted in high levels of intratumoral senescence, as inferred from elevated SAβGal activity, absence of the proliferative marker Ki67, and reduction in phosphorylated Rb (Fig 1C). In a first approach, palbociclib‐treated mice carrying SK‐MEL‐103 xenografts were given a single intravenous injection of GalNP(rho) and fluorescence was analyzed 6 h later. Palbociclib‐treated tumors were strongly autofluorescent (Fig 1D). Importantly, however, rhodamine was detectable above background in mice treated with GalNP(rho), and the signal attributed to rhodamine was higher in tumors treated with palbociclib compared to non‐treated tumors (Fig 1D). Fluorescence was not detected in other organs at 6 h post‐injection, including liver, spleen, and lungs (Appendix Fig S1I and see below Fig 2B). To avoid the detection of autofluorescence, we used beads loaded with indocyanine green (see Appendix Fig S1E), a fluorophore that emits in the far‐red spectrum and therefore is minimally affected by the autofluorescence of palbociclib‐senescent cells. Confirming the rhodamine release data, SK‐MEL‐103 and NCI‐H226 xenografts treated with palbociclib and gal‐encapsulated indocyanine green showed much higher fluorescence compared to tumors treated with a single agent alone (Appendix Fig S1J and K).

### Release of gal‐encapsulated fluorophores in pulmonary fibrosis

Cellular senescence is abundant in pulmonary fibrosis, both in humans and in mice, and actively contributes to the pathological manifestations of this disease (Aoshiba *et al*, 2003, 2013; Hecker *et al*, 2014; Pan *et al*, 2017; Schafer *et al*, 2017). We wondered whether our senescence delivery system would also work in a mouse model of pulmonary fibrosis. Intratracheal instillation of bleomycin in mice produced full‐blown lung fibrosis in a period of 2 weeks, accompanied by focal areas of SAβGal activity and strong collagen deposition as indicated by Masson's trichrome staining (Fig 2A). Two weeks post‐bleomycin administration, mice were intravenously injected with GalNP(rho) and 6 h later fluorescence was measured in the lungs. In this *in vivo* senescence model, autofluorescence was less prominent than in the case of palbociclib‐treated tumors. Importantly, rhodamine release occurred preferentially in fibrotic lungs compared to healthy lungs (Fig 2B). Moreover, confocal microscopy indicated that Rho^+^ cells were more abundant in fibrotic lung lesions compared to non‐fibrotic lungs (Fig 2C).

The differential fluorescence observed between fibrotic and healthy lungs could conceivably reflect, at least in part, a different accessibility and accumulation of the GalNP beads. To evaluate this, we measured the levels of silicon in the lungs and other organs, 6 h after i.v. injection of GalNP beads, by ICP‐MS (inductively coupled plasma mass spectroscopy). Interestingly, the levels of silicon in the lungs and in other tissues were similar between control and bleomycin‐treated mice (Appendix Fig S2A). Therefore, the silica beads reach equally well both healthy and fibrotic lungs (Appendix Fig S2A); however, the release of the fluorophore preferentially occurs within fibrotic lungs (Fig 2B and C). We also wondered if the GalNP beads would retain their activity when administered intratracheally rather than intravenously. Indeed, as in the case of i.v. injection, intratracheal administration of the beads also produced preferential cargo release in fibrotic lungs compared to healthy lungs (Appendix Fig S2B).

Next, we set to characterize in detail the cells targeted by GalNP(rho) in fibrotic lungs using flow cytometry. After excluding endothelial (CD31^+^) and hematopoietic (CD45^+^) cells (Appendix Fig S2C), we quantified the relative number of Rho^+^ cells in double‐negative CD45^−^CD31^−^ cells, which are mostly comprised by lung epithelial cells and fibroblasts. Importantly, bleomycin‐treated lungs showed higher levels of Rho^+^CD45^−^CD31^−^ cells than control lungs (Fig 2D). Further analyses using the epithelial marker EpCAM suggested that the large majority of Rho^+^CD45^−^CD31^−^ cells corresponded to fibroblasts (EpCAM^−^) (Fig 2D). To directly test whether Rho^+^CD45^−^CD31^−^ cells are indeed senescent, CD45^−^CD31^−^ cells from bleomycin‐treated lungs were sorted into Rho^+^ and Rho^−^ subpopulations and subjected to RNAseq. Gene set enrichment analyses (GSEA) using published signatures of senescence (Lasry & Ben‐Neriah, 2015) indicated that Rho^+^CD45^−^CD31^−^ cells present a significant upregulation of senescence signatures (Fig 2E and Appendix Fig S2D and Dataset EV1). We also examined the levels of Rho^+^ cells in endothelial, total hematopoietic cells, lymphocytes, macrophages, and granulocytes. The majority of Rho^+^ cells, both in healthy and fibrotic lungs, were macrophages. However, the relative levels of Rho^+^ macrophages were reduced in bleomycin‐treated lungs, and the same trend was observed in the other cell types (Appendix Fig S2E–G). Although the significance of this reduction in Rho^+^ non‐fibroblastic cells remains to be explored, it could be due to competition by the Rho^+^ fibroblasts present in the bleomycin‐treated lungs. These results demonstrate *in vivo* that GalNP beads release their cargoes within senescent fibroblasts and can be used as a tool to detect and isolate senescent fibroblasts from fibrotic tissues.

### Therapeutic activity of gal‐encapsulated cytotoxic drugs on tumor xenografts

**Figure 3:**
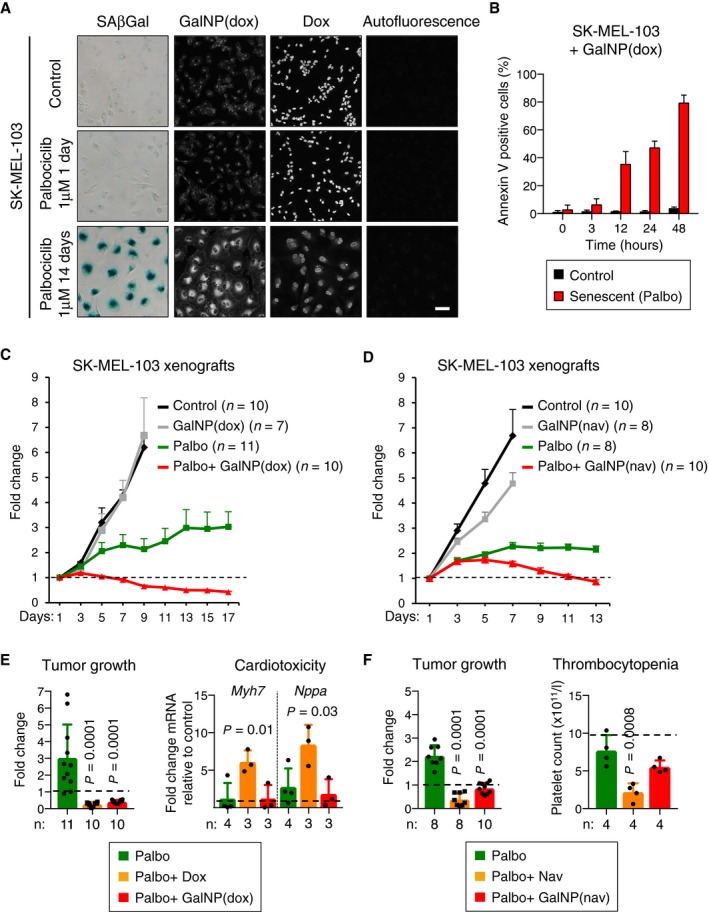
Therapeutic activity of gal‐encapsulated cytotoxic drugs on tumor xenografts ASK‐MEL‐103 melanoma cells were treated with palbociclib (1 μM) for 1 or 14 days, and senescence induction was assessed by SAβgal staining. Next, cultures were exposed to free doxorubicin (50 μM) or GalNP(dox) (1 mg/ml, filtered) for 30 min. Pictures show representative images illustrating doxorubicin fluorescence by confocal microscopy. Scale bar: 50 μm.BSK‐MEL‐103 melanoma cells were treated with palbociclib (5 μM) for 14 days, cultures were exposed to GalNP(dox) (0.06 mg/ml, filtered), and annexin V signal was quantified over time. Representative pictures are shown in Appendix Fig S3F.CAthymic nude female mice carrying subcutaneous SK‐MEL‐103 xenografts were treated daily with palbociclib (oral gavage, 50 mg/kg) and/or GalNP(dox) (tail vein injection, 200 μl of a solution with 4 mg/ml of GalNP containing a total of 1 mg/kg of deliverable doxorubicin), alone or in combination, as indicated. For each tumor, the relative tumor volume change was calculated relative to its baseline prior to treatment. Values are expressed as mean ± SEM. Individual tumor size measurements are shown in Appendix Fig S3G.DSimilar to (B) but using GalNP(nav) (tail vein injection, 200 μl of a solution with 4 mg/ml of GalNP containing a total of 1 mg/kg of deliverable navitoclax), as indicated. Individual tumor size measurements are shown in Appendix Fig S3F.ELeft, fold change of tumor size of SK‐MEL‐103 xenografts, after the indicated daily treatments. Data for palbociclib, and for palbociclib plus GalNP(dox), correspond to the same data in panel (C), at day 17. Data for free doxorubicin (daily tail vein injection, 1 mg/kg, for 17 days) were obtained in parallel. Right, mRNA levels of cardiotoxicity markers in hearts from the same mice. *Actb* and *Gapdh* were used for input normalization. Values are relative to control mice and are expressed as mean ± SD, and statistical significance was assessed by one‐way ANOVA and Dunnett's multiple comparisons test (versus palbociclib‐alone treated group).FLeft, fold change of tumor size, as in (C), after the indicated daily treatments. Data for palbociclib, and for palbociclib plus GalNP(nav), correspond to the same data in panel (D), at day 13. Data for free navitoclax (daily oral gavage, 25 mg/kg, for 13 days) were obtained in parallel. Right, platelet levels in the blood of the same mice. Values are expressed as mean ± SEM in the case of tumor size, and as mean ± SD in the case of platelet counting, and statistical significance was assessed by one‐way ANOVA and Dunnett's multiple comparisons test (versus palbociclib‐alone treated group).

After demonstrating that GalNP beads preferentially release fluorescent cargoes within senescent cells, we wondered whether gal‐encapsulated cytotoxics would also target senescent cells *in vivo*. We screened a panel of 80 anti‐cancer drugs with the aim of identifying drugs with killing activity against both senescent and non‐senescent cells. The killing activity of this panel of drugs was tested in three RB1‐proficient cancer cell lines (SK‐MEL‐103, NCI‐H226, and the hepatocarcinoma cell line Huh7), under normal proliferating conditions or rendered senescent with palbociclib (Appendix Fig S3A–C). We noted that the commonly used cytotoxic drug doxorubicin was among the most efficient cytotoxic drugs against both normal and senescent cells in the three cancer cell lines used. Based on this, we generated gal‐encapsulated beads loaded with doxorubicin [GalNP(dox)] with the aim of preferentially killing senescent cells (Appendix Fig S1E). We took advantage of the intrinsic fluorescence of doxorubicin to assess its preferential release within senescent cells. Addition of GalNP(dox) to senescent SK‐MEL‐103 cells (treated with palbociclib for 14 days) resulted in a strong fluorescent signal after 30 min (Fig 3A). In contrast, growing SK‐MEL‐103 cells showed much weaker fluorescence. As an additional control, we also used SK‐MEL‐103 cells treated with palbociclib for only 1 day, which was not sufficient to induce senescence, but efficiently reduced the levels of phosphorylated RB1 and FOXM1, indicative of CDK4/CDK6 inhibition (Fig 3A and Appendix Fig S3D). Treatment of these short‐term palbociclib‐treated cells with GalNP(dox) resulted in low levels of fluorescence, similar to those of control cells (Fig 3A). To further link drug release with senescence, we performed the same experiment with SAOS‐2 osteosarcoma cells. These cells are null for the *RB1* gene (Li *et al*, 1995) and, therefore, are resistant to palbociclib. Interestingly, treatment of SAOS‐2 cells with GalNP(dox) did not result in doxorubicin release even after long‐term palbociclib treatment (Appendix Fig S3E). It is important to note that the subcellular localization of doxorubicin fluorescence was different depending on its formulation: Free doxorubicin localized in the nucleus, whereas encapsulated doxorubicin rendered perinuclear fluorescence (Fig 3A and Appendix Fig S3E). This reinforces the concept that GalNP beads enter cells through endocytosis and release their cargo in the lysosomal compartment.

It is known that modified forms of doxorubicin with lysosomal tropism efficiently induce apoptosis (Nair *et al*, 2015; Sheng *et al*, 2015). To test whether this was the case in senescent cells treated with GalNP(dox), we measured by time‐lapse fluorescent microscopy the appearance of annexin V‐positive cells. Senescent cells treated with gal‐encapsulated doxorubicin underwent cell death within 48 h, whereas control cells remained viable during this time (Fig 3B and Appendix Fig S3F).

Based on the above date, we decided to test this strategy *in vivo* using gal‐encapsulated doxorubicin and also gal‐encapsulated navitoclax (also known as ABT‐263), which is one of the most efficient senolytic compounds reported to date (Zhu *et al*, 2016; Chang *et al*, 2016; Yosef *et al*, 2016; Pan *et al*, 2017; Appendix Fig S1E). The first model that we used to test senolysis *in vivo* using GalNP(dox) and GalNP(nav) consisted of tumor xenografts treated with palbociclib. Nude mice carrying subcutaneous SK‐MEL‐103 xenografts were treated with daily doses of palbociclib and GalNP(dox), alone or in combination (Fig 3C and Appendix Fig S3G); and a similar experiment was performed with GalNP(nav) (Fig 3D and Appendix Fig S3H). Interestingly, both GalNP(dox) and GalNP(nav) had a clear therapeutic benefit in combination with palbociclib. In contrast, GalNP(dox) and GalNP(nav) had no effect on tumor growth when administered in the absence of palbociclib, demonstrating that their therapeutic activities require the concomitant induction of senescence by palbociclib (Fig 3C and D; and Appendix Fig S3G and H). Similar results were obtained with NCI‐H226 xenografts treated with GalNP(dox) (Appendix Fig S3I). As an additional control, we used empty nanoparticles, GalNP(empty). These empty particles did not have senolytic activity on *in vitro* senescent cells (Appendix Fig S3J), and treatment of mice with GalNP(empty) did not affect the growth of xenografts and did not improve the growth‐inhibitory effect of palbociclib (Appendix Fig S3J).

A potential beneficial aspect of drug encapsulation is reduced drug toxicity. Cardiotoxicity is the most serious side effect of doxorubicin (Chatterjee *et al*, 2010), whereas thrombocytopenia is the main toxicity of navitoclax (Kile, 2014). To assess the effect of gal‐encapsulation on the toxicities of doxorubicin and navitoclax, we selected doses of free and encapsulated drug that were similarly effective in reducing palbociclib‐treated xenografts after daily treatments for ~2 weeks (Fig 3E and F; left panels). For doxorubicin‐treated mice, we measured the mRNA levels of *Myh7* and *Nppa*, which reflect cardiac hypertrophy in response to doxorubicin‐induced cardiomyocyte death (Barry *et al*, 2008; Richard *et al*, 2011). In the case of navitoclax‐treated mice, we measured the levels of serum platelets. Interestingly, gal‐encapsulation completely prevented the cardiotoxicity of doxorubicin (Fig 3E; right panel). Also, gal‐encapsulation of navitoclax produced a mild reduction in platelet counts, which was in contrast to the profound and significant reduction produced by free navitoclax (Fig 3F; right panel). We conclude that gal‐encapsulation of chemotherapeutic drugs is effective in clearing senescent cells *in vivo*, with the added benefit of reducing the toxicities associated with the drugs.

### Therapeutic activity of gal‐encapsulated cytotoxic drugs on pulmonary fibrosis

**Figure 4:**
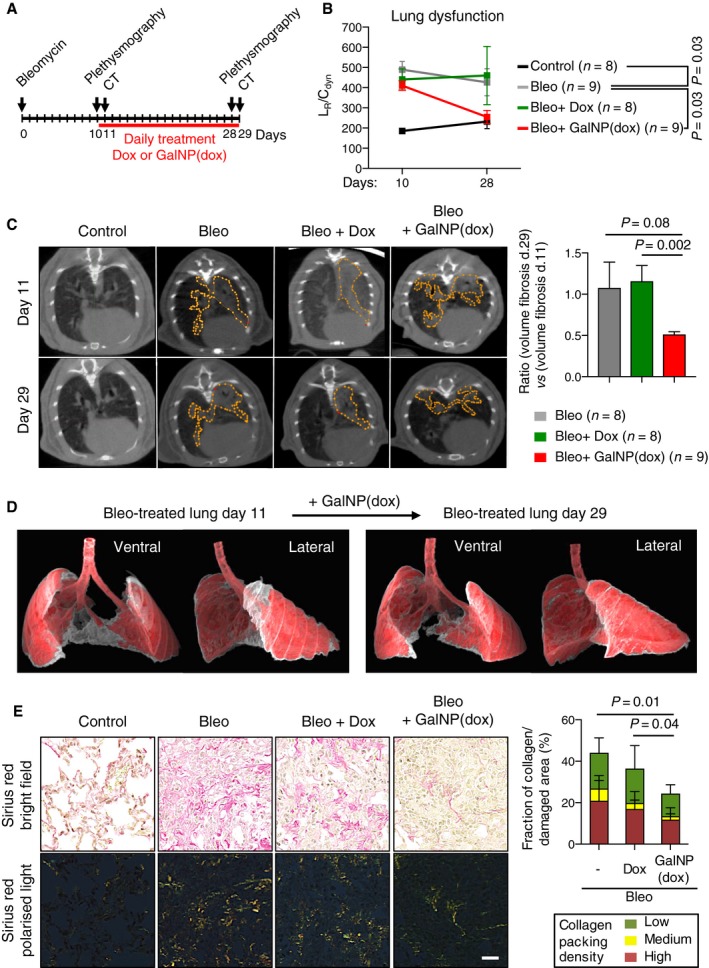
Therapeutic activity of gal‐encapsulated doxorubicin on pulmonary fibrosis AC57BL/6 male mice were subjected to a single intratracheal administration of bleomycin at 1.5 U/kg BW. Beginning at day 10, mice were treated daily with free doxorubicin (tail vein injection, 1 mg/kg) or with GalNP(dox) (tail vein injection, 200 μl of a solution with 4 mg/ml of GalNP containing a total of 1 mg/kg of deliverable doxorubicin), for 18 days, that is, until day 28 post‐bleomycin. Plethysmography and CT were performed at the indicated days.BPlethysmography was used to determine the ratio between lung resistance (L_R_) and compliance dynamics (C_dyn_) in the indicated groups before and after the indicated treatments. Values are expressed as mean ± SEM, and statistical significance was assessed for each group by the two‐tailed Student's *t*‐test comparing the L_R_/C_dyn_ values at day 28.CRepresentative CT images of the indicated treatments at days 11 and 29 post‐bleomycin injury. Each pair of images, at days 11 and 29, correspond to the same mouse. The graph represents the ratio between the volume of fibrosis at end of treatment (day 29) and the volume of fibrosis at the beginning of treatment (day 11). Values are expressed as mean ± SEM, and statistical significance was assessed by the two‐tailed Student's *t*‐test.D3D isocontour‐based volume rendering of a representative lung before and after treatment with GalNP(dox). Healthy lung tissue is shown in red and fibrotic lesions are shown in gray. Ventral and lateral views are shown.ERepresentative images of Sirius red staining of lungs from mice subjected to the indicated treatments 2 days after the last CT analysis, visualized by bright field or polarized light. The graph represents the fraction of collagen per damaged area. Red represents densely packed collagen, yellow represents intermediately packed collagen, and green represents loosely packed collagen. Values are expressed as mean ± SD, and statistical significance comparing the total fraction of collagen per damaged area was assessed by the two‐tailed Student's *t*‐test. Scale bar: 50 μm.

Based on the above data, we decided to test whether gal‐encapsulated doxorubicin could be therapeutic on bleomycin‐induced pulmonary fibrosis. In this disease model, it has been well established that treatment with senolytics reduces fibrosis and favors functional recovery (Pan *et al*, 2017; Schafer *et al*, 2017). We first confirmed that GalNP(dox) efficiently released doxorubicin in lungs from bleomycin‐treated mice, but not in healthy lungs, as determined by total fluorescence (Appendix Fig S4A). Based on this, we performed a longitudinal study evaluating the development of the disease by plethysmography and by computerized tomography (CT) according to the schedule shown in Fig 4A. Bleomycin was administered intratracheally and, when analyzed 10 days later by plethysmography, all the mice presented a significant increase in the L_R_/C_dyn_ ratio (L_R_: lung resistance; C_dyn_: compliance dynamics), which is indicative of pulmonary fibrosis (Fig 4B). Bleomycin‐treated mice were subsequently treated with daily doses of free or gal‐encapsulated doxorubicin (1 mg/kg) for ~2 weeks. Importantly, at the end of the treatment, mice treated with GalNP(dox), but not with free doxorubicin, presented L_R_/C_dyn_ values similar to those of healthy controls (Fig 4B and Appendix Fig S4B). These data were confirmed by computerized tomography, which indicated a significant reduction in the lung volume affected by inflammation and fibrosis in GalNP(dox)‐treated mice (Fig 4C and D and Appendix Fig S4C). Histological analysis of the bleomycin‐treated lungs showed characteristic features of lung fibrosis, namely focal areas of high cellularity with interstitial collagen deposits (stained with Sirius red; Fig 4E). Similar lesions were observed in mice treated with free doxorubicin (Fig 4E). In contrast, mice treated with gal‐encapsulated doxorubicin for ~2 weeks presented a significant collagen reduction in the damaged areas of bleomycin‐treated lungs (Fig 4E). No evidence of hepatic or renal damage was observed in any of the groups, as evaluated by serum markers and histology (Appendix Fig S4D and E).

## Discussion

In recent years, it has become evident that multiple diseases are associated with the presence of senescent cells and that the elimination of these cells has therapeutic benefits in mouse models (Childs *et al*, 2017; Soto‐Gamez & Demaria, 2017). Here, we report a versatile vehicle to deliver small therapeutic compounds to senescent lesions *in vivo*. We show that gal‐encapsulated cytotoxic drugs are therapeutic against tumors treated with senescence‐inducing chemotherapy and against bleomycin‐induced pulmonary fibrosis.

Gal‐encapsulation is based on the high levels of lysosomal β‐galactosidase activity present in many senescent cells (abbreviated as SAβGal). Although SAβGal is not a perfect marker of senescence, damaged or diseased tissues are in general positive for this marker (Sharpless & Sherr, 2015). Gal‐encapsulation consists on spherical particles (100 nm diameter) of porous silica loaded with the chosen therapeutic cargo and then coated with galacto‐oligosaccharides that prevent the diffusion of the cargo out of the silica matrix (Agostini *et al*, 2012). Using this approach, we previously generated gal‐encapsulated rhodamine and we showed preferential release of rhodamine in progeric fibroblasts (dyskeratosis congenita) compared to healthy fibroblasts (Agostini *et al*, 2012). We show here that this is also the case for human cancer cell lines (melanoma, lung squamous cell carcinoma, and head and neck squamous cell carcinoma) responsive to palbociclib. This compound is a CDK4/CDK6 inhibitor that efficiently induces senescence in responsive cancer cells. Importantly, we demonstrate that gal‐encapsulated rhodamine, abbreviated GalNP(rho), preferentially releases rhodamine within senescent lesions *in vivo*. In particular, we demonstrate this in palbociclib‐treated tumor xenografts and in lungs damaged with bleomycin. When we used a different fluorescent cargo, indocyanine green, we also observed preferential release in palbociclib‐treated tumors.

We have used bleomycin‐induced lung fibrosis to investigate which cell types are labeled with rhodamine after *in vivo* treatment with GalNP(rho). Flow cytometry analysis has revealed that lung epithelial cells and fibroblasts are preferentially labeled with rhodamine upon GalNP(rho) injection in bleomycin‐treated lungs compared to control lungs. Interestingly, the RNAseq profile of these cells was enriched in signatures of senescence. Cargo release by GalNP(rho) was also observed in lung macrophages, both in control and bleomycin‐treated lungs. In this regard, it is relevant to mention that macrophages are known to metabolize nanoparticles (Wilhelm *et al*, 2016) and can present SAβGal activity without actually being senescent (Hall *et al*, 2017). Future analyses should determine how gal‐encapsulated drugs affect macrophage populations.

To test the therapeutic potential of our encapsulation method, we first screened a panel of 80 chemotherapeutic agents and we found that doxorubicin was among the most potent agents killing both senescent and non‐senescent cells. In this regard, it is important to point out that doxorubicin kills cells through multiple mechanisms, many of which are not related to cell division (Gewirtz, 1999). In addition, doxorubicin has intrinsic fluorescence thus allowing to track its delivery. Based on this, we generated gal‐encapsulated doxorubicin, or GalNP(dox), and we determined its delivery in cultured cells. As it was the case for rhodamine, gal‐encapsulated doxorubicin was more efficiently released in senescent cells than in control cells. Moreover, this preferential release of doxorubicin by GalNP(dox) resulted in higher levels of apoptosis in senescent cells. Interestingly, we noted that soon after addition of GalNP(dox) to senescent cells, doxorubicin was confined to a perinuclear compartment. This is consistent with the expected route of entry of the nanoparticles via endocytosis. Previous investigators working with conjugated forms of doxorubicin have observed that delivery of doxorubicin into lysosomes is very efficient in inducing apoptosis (Nair *et al*, 2015; Sheng *et al*, 2015). In contrast to the perinuclear localization of encapsulated doxorubicin, administration of the free form of the drug resulted in nuclear localization in all cells, senescent or non‐senescent. These observations support the use of GalNPs as a vehicle to deliver doxorubicin to senescent cells and to induce their apoptotic death.

After validating the use of GalNP(dox) with senescent cultured cells, we focused on the two experimental models mentioned above, namely, bleomycin‐induced lung fibrosis and palbociclib‐induced tumor senescence. Upon administration of GalNP(dox), doxorubicin‐derived fluorescence was higher in bleomycin‐treated lungs compared to control lungs. Bleomycin‐damaged mice were daily treated with intravenous administration of GalNP(dox) or free doxorubucin, from day 10 to day 28 post‐bleomycin. Importantly, gal‐encapsulated doxorubicin significantly improved lung elasticity, as evaluated by plethysmography, while free doxorubicin did not restore lung function. These observations were further supported by the quantification of fibrosis by computerized tomography and by histology.

In addition, GalNP(dox) showed tumor‐regressing activity in combination with palbociclib when administered to mice carrying tumor xenografts (SK‐MEL‐103 melanoma, and H226 lung squamous cell carcinoma). This was also the case when we used encapsulated navitoclax, a well‐established senolytic drug. It is important to note that GalNP(dox) or GalNP(nav) had no effect in the absence of palbociclib, that is, were effective against senescent tumors but not on growing tumors. Also, empty nanoparticles, GalNP(empty), had no effect on tumor size, either alone or in combination with palbociclib. These observations reinforce the concept that gal‐encapsulation is a useful vehicle to deliver therapeutic drugs into senescent tumor cells, but not into non‐senescent tumor cells. The elimination of senescent tumor cells by GalNP‐mediated senolysis may also affect tumor growth by reducing the SASP and its potential pro‐tumorigenic effects. Moreover, the delivery of drugs into senescent cells may have a local bystander effect on neighboring cells and this may enhance its therapeutic effects.

As it is the case of many drugs, doxorubicin and navitoclax present toxicities, which limit their clinical use. The encapsulation of these agents in GalNPs may reduce the exposure of healthy tissues to the drugs and, thereby, diminish undesired effects. In support of this, we have observed that gal‐encapsulation reduced the carditoxicity of doxorubicin and the thrombocytopenia characteristic of navitoclax.

Besides the therapeutic potential of gal‐encapsulation for senescence‐related diseases, our vehicle can be of use for the detection of senescence by *in vivo* imaging. In our work, the encapsulation of fluorophores has allowed us to detect lung tissue regions with senescent cells and palbociclib‐responsive tumors. This opens up the possibility of encapsulating tracers and contrast agents for biomedical imaging of senescent areas that could be of use in multiple age‐related disorders. For example, diagnostic GalNPs could release gadolinium or positron‐emitting radioisotopes in senescent lesions to be detected by MRI or PET, respectively. Gal‐encapsulation could also serve to evaluate the response of solid tumors to the administration of senescence‐inducing chemotherapies or radiotherapies. In addition, cellular senescence is a defining feature of a wide variety of premalignant lesions both in humans and in mice (Collado & Serrano, 2010). Based on this, it is tempting to speculate that GalNPs could also be used for the diagnosis of precancerous tumors. Even more, GalNPs could be potentially employed as a novel theranostic tool, aimed to simultaneously detect and eradicate senescent lesions associated with numerous pathologies, or during aging.

In summary, we contribute a versatile strategy to deliver essentially any small compound to senescent lesions *in vivo*. This novel therapeutic tool can be used to eradicate senescent cells and could be employed in clinical imaging. As a proof of principle, we show that gal‐encapsulated cytotoxic drugs are therapeutically efficient against tumors treated with senescence‐inducing chemotherapy and against pulmonary fibrosis. Finally, and equally important, gal‐encapsulation reduces the systemic toxicities of chemotherapeutic drugs.

## Materials and Methods

### Synthesis of GalNP beads

For the synthesis of the mesoporous silica MCM‐41 scaffolds (Kresge *et al*, 1992), N‐cetyltrimethylammonium bromide (CTAB, Sigma, #H6269) was first dissolved in 480 ml of deionized water at 2.74 mM. Then, 3.5 ml of NaOH 2 M (Sigma, #1310732) was added to the CTAB solution, and temperature was adjusted to 80°C. Next, 5 ml of the polymeric precursor tetraethylorthosilicate (TEOS, Sigma, #131903) was added dropwise to the surfactant solution. The mixture was stirred for 2 h at 80°C, obtaining a white precipitate, referred to as “solid product”. The solid product was centrifuged 13,000 *g* for 20 min and washed with deionized water until reaching a neutral pH, and dried at 60°C, referred to as “NPs as synthesized” (NPs standing for nanoparticles). To obtain the final template‐free NP scaffolds, the “NPs as synthesized” were calcined at 550°C using an oxidant atmosphere for 5 h to remove the CTAB template, thus eliminating any residual surfactant that might affect cell viability, obtaining the “NP scaffolds”.

Loading of the NP scaffolds with the different compounds was performed as follows: For NP(rho), 200 mg of NPs were suspended in a solution of 16 ml of EtOH together with 76.65 mg of rhodamine B (Sigma, #R6226); for NP(icg), 200 mg of mesoporous silica nanoparticles (MSNs) were suspended in a solution of 5 ml of water together with 5 mg of indocyanine green (Sigma, #I2633); for NP(dox), 200 mg of MSNs were suspended in a solution of 12.5 ml of water together with 110 mg of doxorubicin (Carbosynth, #AD15377); for NP(nav), 250 mg of NPs were suspended in 27 ml EtOH together with 91.3 mg of navitoclax (Active BioChem, #A‐1001). These solutions were stirred in round‐bottom flasks for 24 h at room temperature, and solids were isolated by centrifugation at 13,000 *g* for 15 min and dried at 37°C. We refer to these products as “loaded NPs”.

For coating with galacto‐hexasaccharides, the “loaded NPs” were suspended in 7.5 ml of dichloromethane, and 280 μl of 3‐aminopropyltriethoxysilane (APTES, Sigma, #440140) were added to the reaction mixture. After stirring for 5.5 h, solids were isolated by centrifugation at 13,000 *g* for 15 min and dried at 37°C. The obtained solids were suspended in 24 ml of water and 383 mg of Galactan (Carbosynth, #OG71532), mostly (≥ 80%) consisting of six repeating galactose monosaccharides linked through β‐1,4 glycosidic bonds. Stirring was continued at room temperature for 21 h. The final product, referred to as GalNP(cargo), was obtained after several washes with water (cycles of centrifugation at 13,000 *g* for 15 min and suspension in water), followed by drying at 37°C. The GalNP(cargo) were stored in a desiccator at room temperature.

In a few cases, a mixture of β(1,4)‐galacto‐oligosaccharides (Gos) of different lengths (mostly dimers and trimmers) was used to coat the “loaded NPs”, as previously reported (Agostini *et al*, 2012). Briefly, 600 mg of galacto‐oligosaccharide derivative (Gos‐d) were dissolved in 20 ml of EtOH and added to the “loaded NPs”. The suspension was stirred for 5.5 h at room temperature. The final solids were obtained by centrifugation at 13,000 *g* for 15 min and were washed several times with water to eliminate non‐anchored molecules and non‐loaded dye. Finally, solids were dried at 37°C to obtain the final Gos‐coated loaded NPs, abbreviated as GosNP(cargo).

### Characterization of GalNP beads

To characterize the nanomaterials, powder X‐ray diffraction (XRD), thermogravimetric analysis (TGA), elemental analysis (EA), transmission electron microscopy (TEM), N_2_ adsorption‐desorption, and UV‐visible spectroscopy techniques were used. The following equipment was used: X‐ray measurements were performed on a Bruker AXS D8 Advance diffractometer using Cu‐Kα radiation. Thermogravimetric analyses were carried out on a TGA/SDTA 851e Mettler Toledo equipment, using an oxidant atmosphere (Air, 80 ml/min) with a heating program consisting on a heating rate of 10°C/min from 393 K to 1,273 K and an isothermal heating step at this temperature for 30 min. TEM images were taken with a Philips CM10 microscope working at 100 kV. N_2_ adsorption–desorption isotherms were recorded on a Micromeritics ASAP2010 automated sorption analyzer. The samples were degassed at 120°C under vacuum overnight. The specific surface areas were calculated from the adsorption data in the low pressures range using the BET model. Pore size was determined by following the BJH method. UV‐visible spectroscopy was carried out with a Lambda 35 UV/vis spectrometer (Perkin‐Elmer Instruments), and fluorescence spectroscopy was performed with a JASCO spectrofluorometer FP‐8300.

### Cargo release studies

For cargo release studies, 2 mg of GosNP(rho), GosNP(icg), GalNP(rho), GalNP(dox), or GalNP(nav) nanoparticles were suspended in 5 ml of water at pH 4.5, and β‐galactosidase from *Aspergillus oryzae* (Sigma, #G5160) was added at 1,000 ppm (5 mg). Suspensions were stirred, and different aliquots were taken and centrifuged at different time points. The amount of cargo released was determined in a JASCO spectrofluorometer FP‐8300 by monitoring the emission (for rho, icg, dox) or the absorption (for nav) of the dye or drug in the aqueous solution, as a function of time (rho λ_ex_ = 550 nm, rho λ_em_ = 580 nm; icg λ_ex_ = 775 nm, icg λ_em_ = 799 nm; dox λ_ex_ = 495 nm, dox λ_em_ = 556 nm; nav λ_em_ = 356 nm). The same procedure was performed without adding the enzyme to the nanoparticles suspension, as a “blank” control.

### Cell lines

Cell lines SK‐MEL‐103 (human melanoma), NCI‐H226 (human lung squamous cell carcinoma), Huh7 (human hepatocarcinoma), and SAOS‐2 (human primary osteogenic sarcoma) were obtained from ATCC. UT‐SCC‐42B cells (human head and neck squamous cell carcinoma) were kindly provided by Dr. Reidar Grenman (Turku University, Finland). All cell lines were maintained in DMEM, except for NCI‐H226 that were maintained in RPMI, and supplemented with 10% FBS and penicillin–streptomycin (all from Gibco), and incubated in 20% O_2_ and 5% CO_2_ at 37°C. Cells were routinely tested for mycoplasma contamination using the mycoplasma tissue culture NI (MTC‐NI) Rapid Detection System (Gen‐Probe). For senescence induction, cells were supplemented with media containing palbociclib (PD033299, Pfizer Inc.) at 1 or 5 μM, as indicated in the figure legends.

### Cargo release analysis by confocal microscopy

For GalNP(rho) cargo release imaging assays, cells were trypsinized and replated in flat‐bottom μ‐clear 96‐well plates (Greiner Bio‐One, #655087). SK‐MEL‐103 cells were seeded at a density of 6,000 control and 4,000 senescent cells per well. NCI‐H226 and UT‐SCC‐42B cells were plated at a density of 6,000–9,000 control and 10,000–12,000 senescent cells per well. Once cells were attached, culture medium was changed to DMEM supplemented with 0.1% FBS. Three days later, cells were exposed to GalNP(rho) or to GalNP(dox) nanoparticles. The GalNP(rho) and GalNP(dox) were added at a final concentration of ~50 μg/ml in 0.1% FBS‐supplemented DMEM. For cytoplasm and nuclei staining, the cells were incubated for 30 min with 0.05 μM Calcein AM (Mol Probes #C3099) and 4 μM Hoechst (ThermoFisher Scientific, #3342).

For GalNP(dox) cargo release imaging assays, cells were trypsinized and replated in flat‐bottom μ‐clear 96‐well plates (Greiner Bio‐One, #655087). SK‐MEL‐103 cells were seeded at a density of 6,000 control and 4,000 senescent cells per well. Next day, cells were exposed to GalNP(dox) nanoparticles at a concentration of 1 mg/ml in 10% FBS‐supplemented DMEM, previously filtered through a 0.45‐μm syringe filter, for 30 min at 37°C. Then, cells were washed with DMEM and fixed for 10 min with 4% PFA, then washed with PBS and mounted in 30% glycerol.

For cargo release imaging in lung cells, control and bleomycin‐treated mice were tail vein injected with 200 μl of a solution containing GalNP(rho) at 4 mg/ml in DMEM. Mice were sacrificed 6 h post‐injection, and lungs were placed in a cryomold containing OCT (Leica Biosystems) and frozen in dry ice for 30 min. Frozen OCT sections were washed in PBS and fixed in fresh 4% paraformaldehyde for 5 min at room temperature. Coverslips were mounted by using Vectashield Mounting Medium supplemented with 1.5 mg/ml DAPI prior to imaging.

Images were acquired on a TCS‐SP5 (AOBS) laser scanning confocal microscope (Leica Microsystems) using a 20× HCX PL APO 0.7 NA dry objective or a 40× HCX PL APO 1.2 NA oil immersion objective. Rhodamine B was detected by using excitation wavelength of 561 nm (DPSS laser) and with a detection window between 570 and 590 nm. Doxorubicin was detected by using excitation wavelength of 488 nm (argon laser) and with a detection window between 545 and 605 nm. Cells without GalNPs treatments were used in parallel as autofluorescence controls using the corresponding excitation and detection wavelenghts. Images were analyzed with LAS AF v2.6 acquisition software equipped with HCS‐A module (Leica Microsystems). Rhodamine B intensity relative to cell surface was quantified with Definiens XD Developer v2.5 software (Definiens).

### Drug screening assay

For drug screening assays, cells were trypsinized and replated in flat‐bottom μ‐clear 96‐well plates (Greiner Bio‐One, #655087). Control and senescent SK‐MEL‐103 cells were plated at a density of 6,000 and 4,000 cells per well, respectively. Control and senescent Huh7 or NCI‐H226 cells were plated at a density of 7,000 and 15,000 cells per well, respectively. 24 h later, the chemicals from a panel of 80 drugs currently used for the treatment of cancer or in cancer clinical trials were added individually to the cells at a 5 μM final concentration. After 72 h, the cell nuclei were stained with Hoechst (ThermoFisher Scientific, #3342) at 4 μM and dead cells were stained with TO‐PRO‐3 Iodide (Molecular Probes #T3605) at 1 μM.

To assess the number of viable cells, 20 pictures were taken per individual well using an Opera HCS system (Pelkin Elmer) with a 10× objective. Hoechst was detected using excitation wavelength of 360 nm (UV lamp), and TO‐PRO‐3 Iodide was detected using excitation wavelength of 640 nm. Nuclei were analyzed and quantified with Acapella v2.0 software (Perkin Elmer). The data from each single well were averaged, and the number of viable cells was obtained by subtracting the number of TO‐PRO‐3 Iodide positive cells (dead cells) from the number of Hoechst‐positive cells (total cells). The number of viable cells was normalized to the internal control of untreated cells (DMSO only) of each plate.

### Apoptosis assay

For the apoptosis assays, annexin V signal was measured with the IncuCyte Live‐Cell Analysis System (Essen BioScience). Briefly, senescent SK‐MEL‐103 cells (treated with 5 μM palbociclib for 2 weeks) and control SK‐MEL‐103 cells were seeded in flat‐bottom μ‐clear 96‐well plates (Greiner Bio‐One, #655087) in DMEM + 2% FBS. Annexin V Green Reagent for Apoptosis (Cat #4642) was added to the cells. Two hours later, GalNP(dox) was added at a concentration of 0.06 mg/ml, previously filtered through a 0.45‐μm syringe filter. Images were captured over time. Cells with positive signal for annexin V were quantified in triplicates and normalized by the total number of cells per corresponding field.

### Immunohistochemistry on paraffin sections

Tissue samples were fixed in 10% neutral buffered formalin (4% formaldehyde in solution), paraffin‐embedded, and cut into 3‐mm sections, which were mounted and dried. For different staining methods, slides were deparaffinized in xylene and re‐hydrated through a series of graded EtOH until water. Masson's trichrome and Sirius red stainings were used to assess the presence of fibrotic areas in the lungs, as indicated. Hematoxylin and eosin (HE) staining was used to analyze liver damage, as indicated. For immunohistochemistry, an automated immunostaining platform was used (Ventana discovery XT, Roche). Antigen retrieval was first performed with high pH buffer (CC1m, Roche), endogenous peroxidase was blocked, and slides were then incubated with the appropriate primary antibodies as detailed: Ki67 (Master Diagnostica, #0003110QD), phosphorylated Rb (Ser807/811) (Cell Signalling Technology, #9308). After incubations with the primary antibody, slides were incubated with the corresponding secondary antibodies and visualization systems (OmniRabbit, Ventana, Roche) conjugated with horseradish peroxidase (Chromomap, Ventana, Roche). Immunohistochemical reactions were developed by using 3,3′‐diaminobenzidine tetrahydrochloride (DAB) as a chromogen, and nuclei were counterstained with hematoxylin. Finally, the slides were dehydrated, cleared, and mounted with a permanent mounting medium for microscopic evaluation.

### Animal models

All mice were maintained at the Spanish National Cancer Research Centre (CNIO) under specific pathogen‐free conditions in accordance with the recommendations of the Federation of European Laboratory Animal Science Associations (FELASA). Mice were housed in ventilated racks with integration of Individually Ventilated Caging (IVC) units in the building ventilation systems. All animal experiments were approved by the Ethical Committee for Research and Animal Welfare (CEIyBA) and performed in accordance with the guidelines stated in the International Guiding Principles for Biomedical Research Involving Animals, developed by the Council for International Organizations of Medical Sciences (CIOMS).

### Xenograft formation

Tumor xenografts were established using SK‐MEL‐103 or NCI‐H226 cell lines. Cells were trypsinized, counted with a haemocytometer, and injected subcutaneously (10^6^ cells in a volume of 100 μl per dorsolateral flank) in 8‐ to 10‐week‐old athymic nude female mice (Hsd:Athymic Nude‐Foxn1nu) purchased from Envigo. Tumor volume was measured every 2 days with a caliper and calculated as *V* = (*a* × *b* ^2^)/2 where *a* is the longer and *b* is the shorter of two perpendicular diameters.

### Fibrosis

To induce pulmonary fibrosis, 8‐ to 10‐week‐old male C57BL/6 wild‐type mice were anesthetized by intraperitoneal injection with a mix containing ketamine (75 mg/kg) and medetomidine (1 mg/kg). The animals were placed on a Tilting WorkStand for rodents (EMC Hallowell) and intubated intratracheally with a 24GA catheter (BD Biosciences). Then, bleomycin (Sigma, #15361) was intratracheally inoculated in at 1.5 U/kg of body weight. Serial sections of the lung were stained with Sirius red to assess the presence of collagen. Whole slides were digitalized with a slide scanner (Mirax Scan, Zeiss) using polarized light, and images were acquired with the Pannoramic Viewer Software (3DHISTECH). Image analysis and quantification of colored signals (red, yellow, and green) within damaged areas was performed in a completely automated manner using the AxioVision software package (Zeiss). At least seven mice per group were quantified.

### Mouse treatments

*In vitro* experimental calculations indicated that approximately ~30 mg of drug, both doxorubicin and navitoclax, are deliverable per g of GalNPs (see Appendix Table S3). GalNPs were weighted in 8 ml Wheaton glass vials (Sigma, Z256161) at 4 mg/ml in DMEM supplemented with 10% FBS and stirred with a magnetic stir bar for 1 h. Mice were i.v. injected as indicated, with 200 μl of a nanoparticle solution containing 4 mg/ml (equivalent to 1 mg/kg of deliverable drug). Doxorubicin (Sigma, #D1515) was dissolved in 7% saline/DMEM solution at 1 mg/kg and administered by daily i.v. (tail vein) injection as indicated. Navitoclax, also known as ABT263 (Active Biochem, #A‐100), was administered by daily oral gavage, for 13 days, at 25 mg/kg, dissolved in 15% DMSO/PEG400. Palbociclib (Pfizer Inc.) was dissolved in 50 mM sodium lactate at 12.5 mg/ml and administered by daily oral gavage at the indicated doses. Palbociclib treatment started 1 day before GalNPs or free drugs administration.

### Flow cytometry

Mice were anesthetized by intraperitoneal injection with a mix containing ketamine (75 mg/kg) and medetomidine (1 mg/kg). Mouse lungs were intracardially perfused with 20 ml ice‐cold PBS, removed, and intratracheally inoculated with 2 ml of 5 U/ml dispase (Corning). After 10‐min incubation at 37°C, lungs were cut into small pieces with surgical blades (Braun) and transferred to GentleMacs M Tubes (Miltenyi) containing 7 ml HBSS, 1% FBS, 20 U/ml DNase I (Promega), and 70 U/ml collagenase I (Gibco). Samples were homogenized in a GentleMacs Dissociator (Miltenyi) using the “mouse lung 01” program, incubated for 30 min at 37°C under shaking, and re‐homogenized with the “mouse lung 02” GentleMacs Dissociator program. The homogenized mixture was subsequently filtered through 70 and 40 nm cell strainers (BD Biosciences) and centrifuged at 300 *g* for 5 min. The cell pellet was resuspended in 2 ml erythrocyte lysis buffer (Qiagen) for 4 min at RT for red blood cell lysis. Cells were then resuspended in 13 ml DMEM + 10% FBS. Cells were counted and viability was assessed using trypan blue (Sigma) in an automated cell counter Countess (Molecular Probes), and resuspended at 10^7^ cells/ml in HBSS + 2% FBS containing 10 mM HEPES buffer (Gibco). The following rat monoclonal conjugated antibodies were used: anti‐CD31 APC (1:400, BD Biosciences; #551260), anti‐CD45 APC (1:200, BD Biosciences, #559864), anti‐CD326 (EpCAM) AF488 (1:200, Biolegend, #118210), anti‐CD45.2 APC‐Cy7 (1:100, Biolegend, #109824), anti‐F4/80 BV421 (1:50, Biolegend, 123131), anti‐CD11b PE‐Cy7 (1:200, BD Biosciences #552850). DAPI (Sigma) or forward and side scatter (FSC/SSC) was used for live/dead discrimination. All samples were acquired and sorted using an InFlux (BD) equipped with 640, 561, 488 and 355 nm lines. OneComp Beads (eBioscience) were used as compensation controls to assess and correct spectral overlapping. Population gating was performed using fluorescence minus one (FMO) controls (Appendix Fig S2C, E and F). Pulse processing was used to exclude cell aggregates, and at least 50,000 events were collected for analysis.

### RNAseq

Total RNA from flow‐sorted lung cells was extracted with an RNAeasy mini kit (Qiagen, #74106). ~2 ng of total RNA samples (range 0.4–5 ng) was processed with the SMART‐Seq v4 Ultra Low Input RNA Kit (Clontech) by following manufacturer instructions. Resulting cDNA was sheared on a S220 Focused‐ultrasonicator (Covaris) and subsequently processed with the “NEBNext Ultra II DNA Library Prep Kit for Illumina” (NEB #E7645). Briefly, oligo(dT)‐primed reverse transcription was performed in the presence of a template switching oligonucleotide; double‐stranded cDNA was then produced by 14 cycles of PCR and submitted to acoustic shearing. Fragments were processed through subsequent enzymatic treatments of end‐repair, dA‐tailing, and ligation to Illumina adapters. Adapter‐ligated libraries were completed by limited‐cycle PCR (eight cycles). The resulting purified cDNA libraries were applied to an Illumina flow cell for cluster generation and sequenced on an Illumina HiSeq2500 by following manufacturer's protocols. Image analysis, per‐cycle basecalling, and quality score assignment were performed with Illumina Real Time Analysis software. Conversion of Illumina BCL files to bam format was performed with the Illumina2bam tool (Wellcome Trust Sanger Institute—NPG). Sequencing quality was analyzed with FastQC; reads were aligned to the mouse genome (GRCm38/mm10) using TopHat‐2.0.10 (Trapnell *et al*, 2012), Bowtie 1.0.0 (Langmead *et al*, 2009) and Samtools 0.1.19.0 (Li *et al*, 2009); and transcripts assembly, abundances estimation, and differential expression were calculated with DESeq2 (Love *et al*, 2014). The estimated significance level (*P*‐value) was corrected to account for multiple hypotheses testing using Benjamini and Hochberg False Discovery Rate (FDR) adjustment. Genes with FDR ≤ 0.05 were considered differentially expressed genes. GSEA of differentially expressed genes was performed using the GSEA software v2.0.6 and obtained from the Broad Institute and following the developer's protocol. The previously obtained differentially expressed genes were ranked according to their *t*‐statistic. This ranked file was used as input for the enrichment analysis. All basic and advanced fields were set to default and only gene sets significantly enriched at a FDR *q*‐values < 0.25 were considered.

### Analysis of mRNA levels

For lung samples, total RNA from flow‐sorted lung cells was extracted with an RNAeasy mini kit (Qiagen, #74106). For heart samples, total RNA was isolated by acid guanidinium thiocyanate–phenol–chloroform extraction. Up to 4 μg of total RNA was reverse transcribed into cDNA using iScriptTM Advanced cDNA Synthesis Kit for RT–qPCR (Bio‐Rad #172‐5038). For qRT–PCR, each cDNA sample was assayed in triplicate reactions in optical‐grade, 386‐well plates (Applied Biosystems). Each reaction contained 6 μl of SybrGreen (2×) (Applied Biosystems), 2 μl of cDNA template, 3.4 μl of DNase‐free water, and 0.6 μl of primers (10 μM f.c.). All PCR runs were performed on a QuantStudio™ 6 Flex Real‐Time PCR System using QuantStudio™ 6 and 7 Flex Real‐Time PCR software v1.0 (Applied Biosystems). Reaction conditions were as follows: 10 min at 95°C followed by 40 cycles of 15 s at 95°C, 1 min at 60°C, and 1 min at 62°C. The housekeeping genes *Actb* and/or *Gapdh* were used for input normalization, as indicated.

List of primers used for mRNA expression analyses:

*Actb* Forward: 5′‐GGCACCACACCTTCTACAATG‐3′
*Actb* Reverse: 5′‐GTGGTGGTGAAGCTGTAGCC‐3′
*Gapdh* Forward: 5′‐TTCACCACCATGGAGAAGGC‐3′
*Gapdh* Reverse: 5′‐CCCTTTTGGCTCCACCCT‐3′
*Il6* Forward: 5′‐GTTCTCTGGGAAATCGTGGA‐3′
*Il6* Reverse: 5′‐GGTACTCCAGAAGACCAGAGGA‐3′
*Myh7* Forward: 5′‐TGCAGCAGTTCTTCAACCAC‐3′
*Myh7* Reverse: 5′‐TCGAGGCTTCTGGAAGTTGT‐3′
*Nppa* Forward: 5′‐ATCTGCCCTCTTGAAAAGCA‐3′
*Nppa* Reverse: 5′‐ACACACCACAAGGGCTTAGG‐3′

### Western blot

Cells were harvested after treatment with the indicated compounds in NET buffer (150 mM NaCl, 50 mM Tris pH 8, 2 mM EDTA, 1% Triton, 0.1% FBS). Identical amounts of whole lysates (30 μg) together with a Chameleon™ Duo pre‐stained protein ladder (LI‐COR, #928‐60000) were resolved in 4–12% Bis‐Tris gels (NuPAGE Invitrogen, #NP0321BOX) and transferred to Amersham Protran 0.2 μm nitrocellulose membranes (GE Healthcare, #10600001). Blots were incubated with the primary antibodies rabbit anti‐P‐Rb S887/811 (1:1,000, Cell Signaling, #8516), rabbit anti‐FoxM1 D1205 (1:5,000, Cell Signaling, #5436), rabbit anti‐ACTB GTU‐88 (1:10,000, Sigma, #T6557), and goat anti‐p21 C‐19‐G (1:200, Santa Cruz, #sc‐397‐G) at 4°C overnight, and subsequently incubated with the corresponding secondary anti‐IgG antibodies anti‐rabbit IRDye 680 CW (1:15,000, LI‐COR, #926‐68071), anti‐rabbit IRDye 800 CW (1:15,000, LI‐COR, #926‐32211), and anti‐goat IRDye 680 CW (1:10,000, LI‐COR, #926‐68074) for 1 h at room temperature. Blots were analyzed with an Odyssey Fc imaging system (LI‐COR).

### SAβGal activity assay

SAβGal staining was performed in cells, whole tissues (either lungs or tumor xenografts) or in tissue cryosections (either lungs or tumor xenografts) preserved in OCT using the Senescence β‐Galactosidase Staining Kit (Cell Signaling). In cells, SAβGal staining was performed following the manufacturer's instructions. Briefly, whole tissues or tissue cryosections were fixed at room temperature, for 45 min or 2 min, respectively, with a solution containing 2% formaldehyde and 0.2% glutaraldehyde in PBS, washed three times with PBS, and incubated overnight at 37°C with the Staining Solution containing X‐gal in N‐N‐dimethylformamide (pH 6.0). In the case of whole tissue staining, tissues were subsequently dehydrated with two consecutive steps in 50 and 70% EtOH and embedded in paraffin for serial sectioning. Sections were counterstained with nuclear fast red (NFR) or were processed for immunohistochemistry.

### Lung *in vivo* imaging by CT

The acquisition was made on a CT Locus high‐resolution computed tomography system (GE Healthcare) specifically designed for small laboratory animals and with an isotropic resolution of 45 μm. Mice were anesthetized with a 4% rate of isoflurane (IsoVet Braun) during the induction, and 2% during the maintenance period (scanning time). Micro‐CT image acquisition consisted of 400 projections collected in one full rotation of the gantry during ~10 min in a single stage focused on the lungs, with a 450‐mA/80‐kV X‐ray tube. The resulting raw data were reconstructed to a final image volume of 875 × 875 × 465 slices at (93 μm) 3 voxel dimensions. CT images were analyzed with Microview 2.2 software (GE Healthcare). To measure the volume of the fibrotic areas, a region of interest (ROI) was drawn manually over the lung “slice by slice” on the areas that showed abnormal increase of density corresponding to fibrotic tissue. Finally, a three‐dimensional ROI was generated providing the fibrotic volume. Image analysis quantification was performed by an independent and well‐trained technician in a blinded manner. 3D isocontour‐based volume rendering was performed using AMIDE software. All procedures were carried out according to the European Normative of Welfare and Good Practice (2010/63/UE).

### Plethysmography

Mice were anesthetized by intraperitoneal injection with a mix containing ketamine (75 mg/kg) and medetomidine (1 mg/kg). Animals were placed on a Tilting WorkStand for rodents (EMC Hallowell) and intubated intratracheally with a 24GA catheter (BD Biosciences). Then, animals were placed in a plethysmograph adapted for anesthetized rodents (emka TECHNOLOGIES). A MiniVent Model 845 (Harvard Apparatus) connected to the plethysmograph was used to ventilate the mice at a frequency of 150 beats/min and 10 ml/kg of tidal volume. Transpulmonary pressure was measured with a pressure transducer connected to the cannula. Airflow and tidal volume were determined using a flow transducer fixed to the plethysmograph chamber. Lung resistance (L_R_) and compliance dynamics (C_dyn_) were calculated and analyzed with iox2 software (emka TECHNOLOGIES).

### *In vivo* and *ex vivo* imaging by IVIS

An IVIS Spectrum Imaging System (Perkin Elmer Inc) was used for *in vivo* and *ex vivo* fluorescence imaging. For *in vivo* imaging, mice were anesthetized with a 4% rate of isoflurane (IsoVet Braun) during the induction, and 2% during the maintenance period (scanning time). For *ex vivo* imaging, mice were sacrificed by CO_2_ exposure in a euthanasia chamber, and organs and tumor xenografts were analyzed immediately after harvesting. Rhodamine B was detected using excitation wavelength of 535 nm and emission wavelength of 580 nm. Indocyanine green was detected using excitation passband of 710–760 nm and emission passband of 810–875 nm. Doxorubicin was detected using excitation wavelength of 430 nm and emission wavelength of 600 nm. Fluorescence imaging quantification was performed by Living Image 3.2 software (Perkin Elmer Inc). A region of interest area (ROI) was drawn over the fluorescent signal either in lung or xenograft tumor samples in order to quantify the release of the fluorophores. Fluorescence activity is measured in photons per second per square centimeter per steradian (p/s/cm^2^/sr).

### Blood analysis

Blood samples were obtained by sub‐mandibular blood extraction and collected in EDTA‐coated tubes (Aquisel). Platelet counts were measured with an Automated Blood Cell counter (Abbacus). For hepatic and renal function assessment, blood samples were centrifuged at 1,700 *g* for 5 min, and the serum (supernatant) was removed. Serum samples were added at a volume of 50 μl into VetScan^®^ Mammalian Liver Profile reagent rotors (Abaxis, #500‐1040). Hepatic and renal profiles were assessed by analysis of the rotors with a VetScan^®^ Chemistry Analyzer (Abaxis).

### Silica biodistribution

Mice were sacrificed 6 h post‐GalNP(rho) injection, and selected organs were extracted (lung, liver, spleen, bladder, kidney, heart, gut, and tail) for Si detection. The extracted organs were first weighed and then digested with 1 ml of tetramethylammonium hydroxide solution 25% in H_2_O (TMHA, Sigma, #75592). Digestion was carried out in closed polytetrafluoroethylene (PTFE) vessels for 2 h at 80°C, using a digestion unit Bloc digest 20 (Selecta). After cooling, digested samples were diluted to 10 ml in polypropylene Erlenmeyer flasks (in order to avoid Si contamination) and kept in polystyrene tubes. For the analysis, 0.5 ml of the digested samples was diluted to 10 ml with a solution of 2% nitric acid and 1% hydrochloric acid. Standard solutions were prepared from silicon standard (1,000 mg/l Si in NaOH, Sigma #08729) for Inductively Coupled Plasma Mass Spectroscopy (ICP‐MS), and were digested and treated exactly the same way as the samples. Samples were analyzed with an Inductively Coupled Plasma Mass Spectrometer System (ICP‐MS) Agilent 7900. Results were calculated using the calibration with digested standard solutions. Data are expressed as μg Si/g sample.

**The paper explained**

**Problem**

Senescence is a cellular program that is triggered by multiple types of damage. Senescent cells are present in many diseases and accumulate in multiple tissues during aging, thereby participating actively in tissue deterioration. A major goal in this area is to find ways to eliminate senescent cells within tissues. Senescent cells share a number of features, including a very low or inexistent capacity to proliferate, and a robust secretion of extracellular factors that can severely modify the microenvironment. The active secretory machinery of senescent cells is associated with a high abundance of lysosomes, and thereby, senescent cells are characterized by high levels of lysosomal β‐galactosidase activity. We have used this trait to deliver drugs preferentially into senescent cells.

**Results**

We have designed a drug encapsulation system that uses galacto‐oligosaccharides to coat a silica scaffold containing the drug of interest. The release of gal‐encapsulated drugs requires digestion by the lysosomal β‐galactosidase. Senescent cells are more efficient than non‐senescent cells in digesting and releasing gal‐encapsulated drugs. We demonstrate that gal‐encapsulated drugs are preferentially released into damaged tissues containing senescent cells, such as bleomycin‐induced lung fibrosis. The release of gal‐encapsulated doxorubicin into fibrotic lungs results in a remarkable reduction of fibrosis and in recovery of respiratory function. In contrast, free doxorubicin does not ameliorate pulmonary fibrosis. In the case of xenograft tumors, treatment with chemotherapy often triggers senescence in tumor cells, as it is the case of palbociclib. Co‐treatment of tumors with palbociclib and gal‐encapsulated drugs results in a more efficient tumor regression. Gal‐encapsulation has the additional advantage of reducing the exposure of non‐target organs to the drugs.

**Impact**

This study presents proof of principle for the biological activity of a versatile encapsulation method that allows to deliver drugs preferentially in diseased tissues containing senescent cells. This may open new therapeutic opportunities for severe diseases, such as pulmonary fibrosis. The delivery of drugs into senescent cells may also improve cancer chemotherapy by eliminating chemotherapy‐induced senescent cells. Finally, it may have applications in the diagnosis of senescence‐associated diseases.

### Statistical methods

In cell culture experiments, at least three independent replicates were assayed to ensure reproducibility. In animal experiments, sample sizes for comparisons between treatments followed Mead's recommendations (Festing *et al*, 2002). In particular, the accumulated n value (N) for a given comparison minus the number of groups or treatments (T) was between 10 and 20 in most of the experiments, as recommended. In the case of bleomycin‐induced pulmonary fibrosis experiments, the n value was higher due to the intrinsically high variability of the assay. For *in vivo* studies, mice were excluded following pre‐established criteria if they developed humane‐intervention end‐point complications in the course of the study. All mice were age and weight matched. No additional randomization was used for animal studies. For cell culture fluorescence analysis, quantifications were performed in a blinded manner. For plethysmography measurements and CT analysis, the evaluators were also blind to the treatments. For the xenograft assays, investigators were not blinded to the experimental groups. Statistical tests were performed only on experiments with at least three replicates. Statistical significance was assessed as appropriate using either the Student's *t*‐test (two‐tailed, paired or unpaired), the Fisher exact test, or the one‐way ANOVA followed by a post‐hoc Dunnett's multiple comparisons test, as indicated in the figure legends. Data were tested for normal distribution using the Shapiro–Wilk test and for equal variance using the *F*‐test. Normal distribution and equal variance was confirmed in the large majority of data, and therefore, normality and equal variance was assumed for all samples. Data were represented as the mean of the indicated experimental replicates. Error bars represent the standard deviation (SD) or the standard error of the mean (SEM), as indicated in the figure legends. *P*‐values inferior to 0.0001 were assigned a value of *P* = 0.0001. *P*‐values are indicated in the figures.

### Data availability

The primary RNAseq data from this publication have been deposited to the GEO Omnibus database (https://www.ncbi.nlm.nih.gov/geo) and assigned the identifier GSE97685.

## Author contributions

DM‐E and MR performed most of the experiments, contributed to experimental design, data analysis, discussion, and writing; IG, CG, BL‐T, AB prepared and characterized the encapsulated drugs; MP‐R performed the *in vitro* apoptosis assays; SC and SL performed the drug screening, some of the experiments with *in vitro* cultured cells, and some of the *in vivo* assays with fluorophores in xenografts; MM‐M supervised animal experimentation and performed most of the animal manipulations; ACU helped with the plethysmography assays; GG and FM performed the lung computed tomography analysis; SD, TV, and DJS contributed to the experimental design, data analysis, discussion, and writing; JMR and RM‐M designed and supervised the generation of the encapsulated drugs, and contributed to data analysis, discussion and writing; MS designed and supervised the biological studies, analyzed the data, and wrote the manuscript. All authors revised and commented on the manuscript.

## Conflict of interest

S.D., T.V., and D.J.S. are employees of Pfizer Inc. and hold shares in the company. J.R.M., R.M.‐M., and M.S. are founders of Senolytic Therapeutics, S.L. (Spain) and Senolytic Therapeutics, Inc. (USA) aimed at developing senolytic therapies.

## Supporting information



Appendix

Dataset EV1

Review Process File

Source Data for Appendix
