# Supplementary material for: A versatile drug delivery system targeting senescent cells
Source: EMBO Mol Med. 2018 Jul 16;10(9):e9355. doi: 10.15252/emmm.201809355 (PMC6127887; doi:10.15252/emmm.201809355)
Supplement: Supplementary file 1 — Appendix [file EMMM-10-e9355-s001.pdf]

Appendix

A versatile drug delivery system targeting senescent cells

Daniel Muñoz-Espín, Miguel Rovira, Irene Galiana, Cristina Giménez, Beatriz Lozano-Torres, Marta Paez-Ribes, Susana Llanos, Selim Chaib, Maribel Muñoz, Alvaro C. Ucero, Guillermo Garaulet, Francisca Mulero, Stephen Dann, Todd VanArsdale, David J. Shields, Andrea Bernardos, José Ramón Murguía, Ramón Martínez-Máñez and Manuel Serrano

Contents:

Appendix Figures S1 to S4
Appendix Tables S1 to S3

A

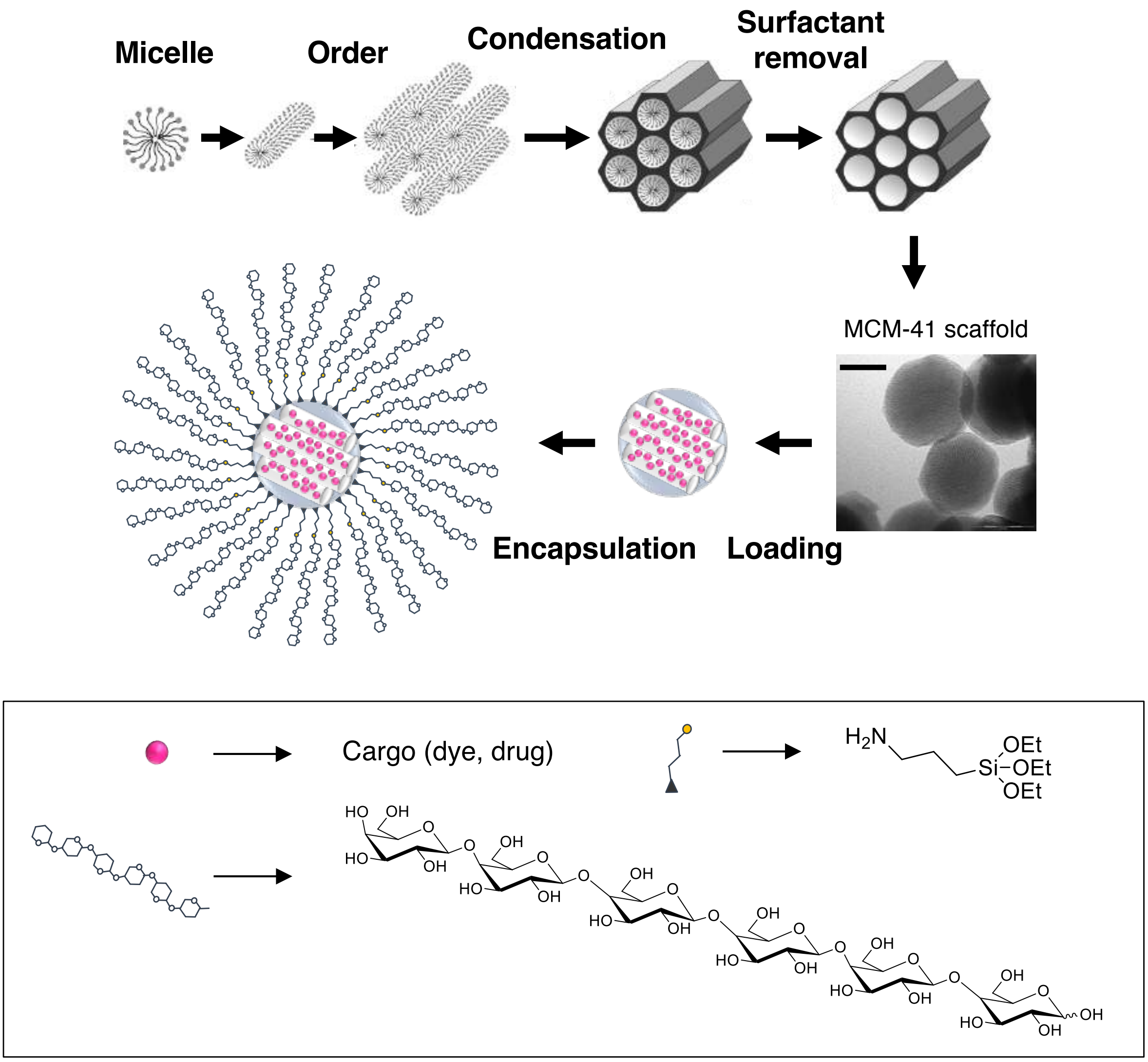
Micelle
Order
Condensation
Surfactant removal
MCM-41 scaffold
Encapsulation
Loading
Cargo (dye, drug)
H2N
OEt
Si
OEt
OEt

B

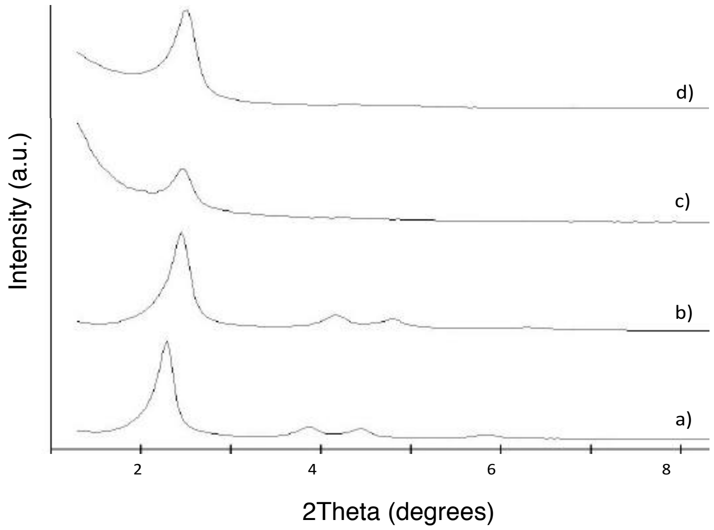
Intensity (a.u.)
2Theta (degrees)
2
4
6
8
a)
b)
c)
d)

C

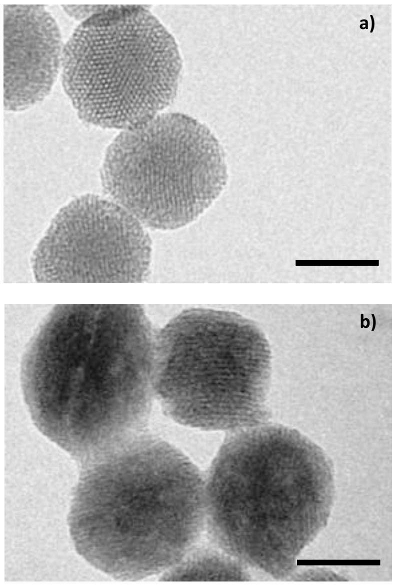
a)
b)

D

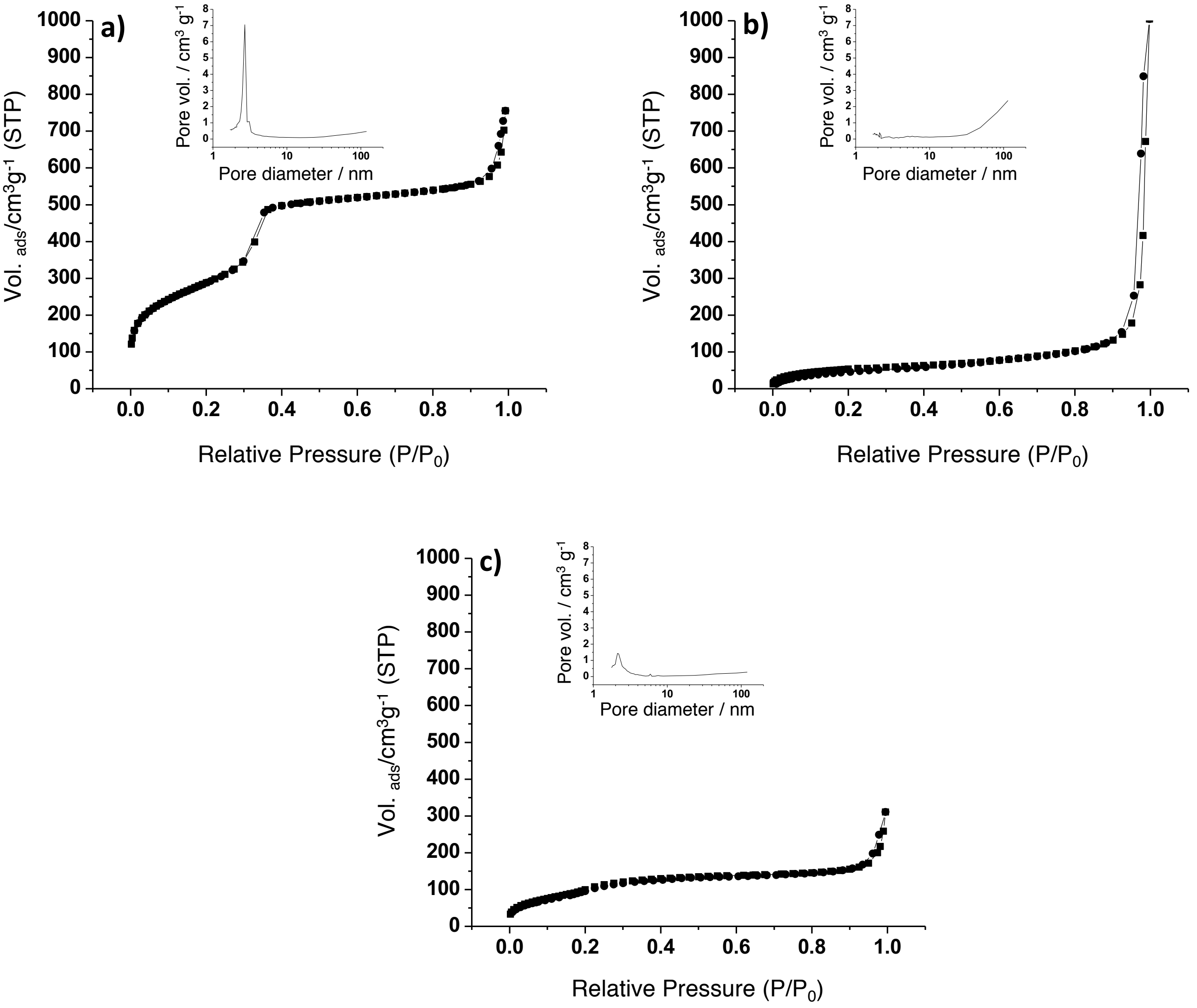
a)
b)
c)
Vol. ads/cm3g-1 (STP)
Relative Pressure (P/P0)
Pore vol. / cm3 g-1
Pore diameter / nm

E

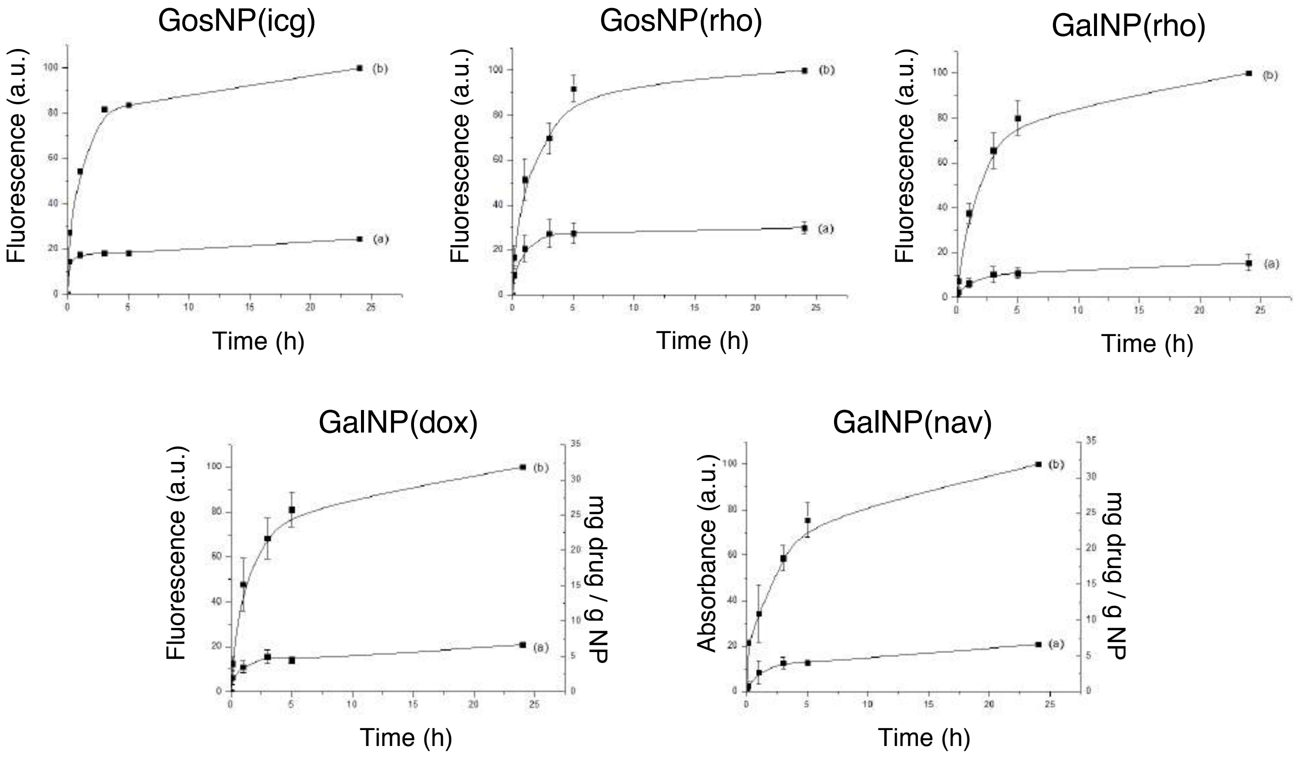
GosNP(icg)
GosNP(rho)
GalNP(rho)
GalNP(dox)
GalNP(nav)
Fluorescence (a.u.)
Absorbance (a.u.)
mg drug / g NP
Time (h)
(a)
(b)

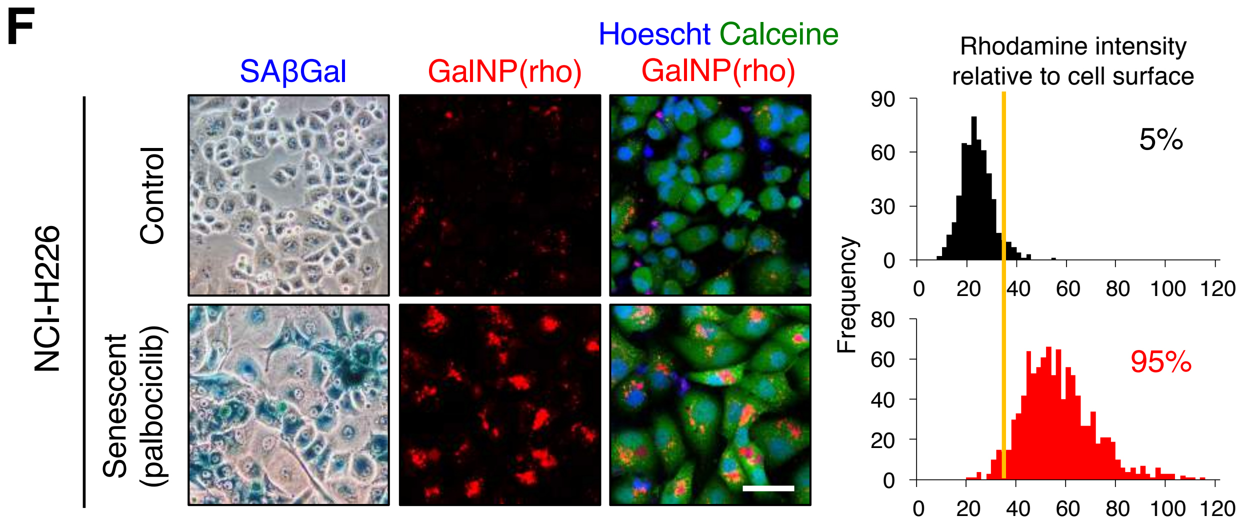
F
NCI-H226
Control
Senescent (palbociclib)
SAβGal
GalNP(rho)
Hoescht Calceine GalNP(rho)
Rhodamine intensity relative to cell surface
Frequency
5%
95%

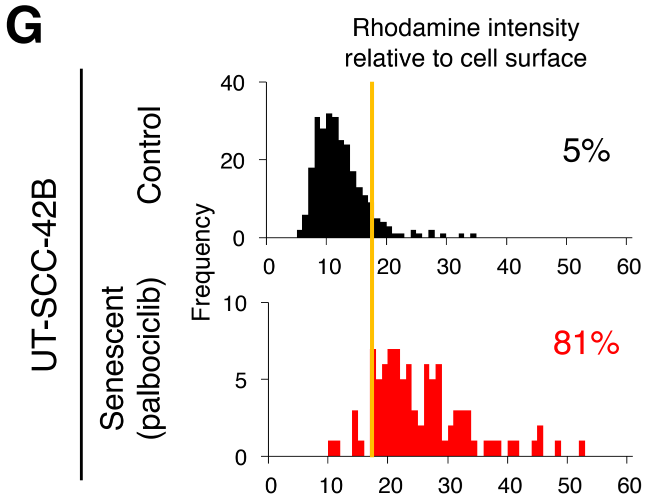
G
UT-SCC-42B
Control
Senescent (palbociclib)
Rhodamine intensity relative to cell surface
Frequency
5%
81%

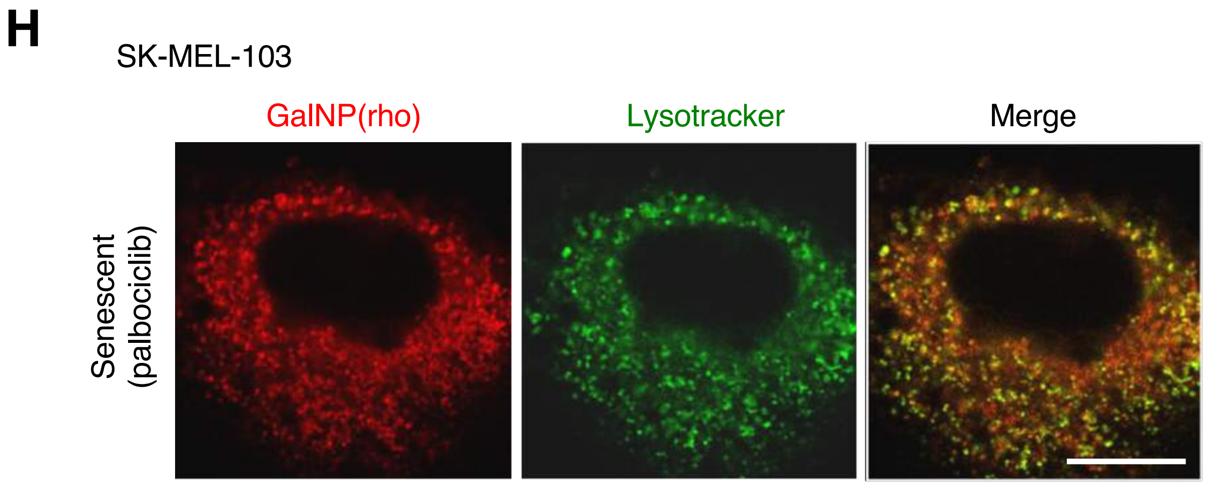
H
SK-MEL-103
GalNP(rho)
Lysotracker
Merge
Senescent (palbociclib)

Appendix Figure S1
(page 4 out of 4)

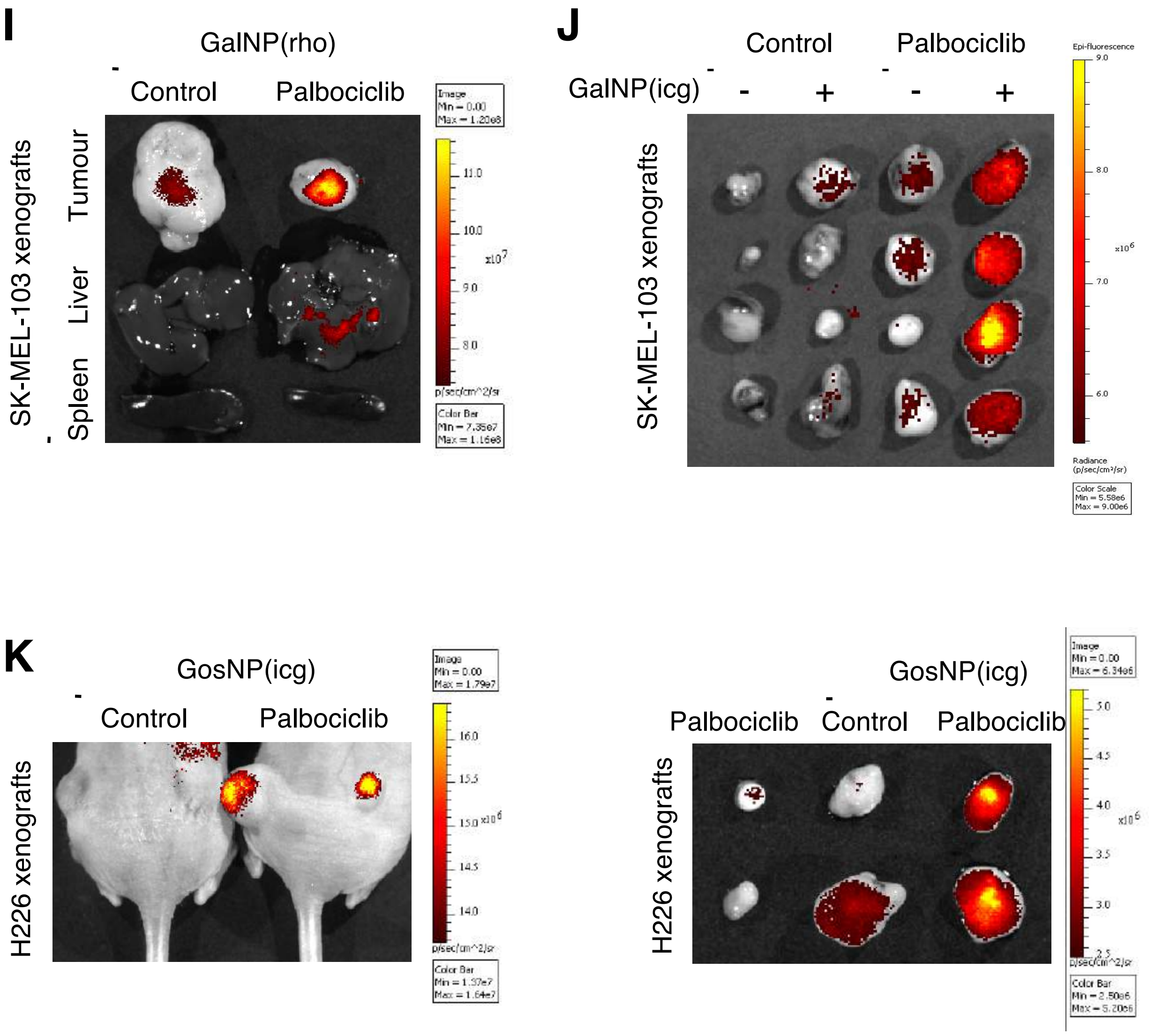

Appendix Figure S1. Release of gal-encapsulated fluorophores in xenografts

A, Schematic representation of the synthesis of the GalNP beads. First, a super-micellar-template is formed in basic water solution. Next, the inorganic siliceous precursor tetraethylortosilicate hydrolyses and condensates of around the template. The final mesoporous inorganic MCM-41 scaffold, which presents cylindrical unidirectional empty channels arranged in a hexagonal distribution, is obtained by the removal of the surfactant template by calcination. Next, the cargo is loaded in the mesopores and, finally, the beads are encapsulated with 6-mer galacto-oligosaccharides that will act as molecular gate to obtain the final GalNP system. Scale bar: 50 nm

B, Powder X-ray patterns of solids MSNs as-synthesized, NPs calcined, GosNP(rho) and GalNP(rho). The XRD of NPs as-synthesized (curve a) shows the typical four low-angle reflections of a hexagonal ordered array indexed as (100), (110), (200), and (210) Bragg peaks. In curve b, corresponding to the NPs calcined sample, a significant shift of the (100) reflection in the XRD is clearly observed. This displacement, together with the broadening of the (110) and (200) reflections, is consistent with an approximate cell contraction of ca. 6-8 Å and attributed to the condensation of silanols during the calcination step. XRD patterns for solids GosNP(rho) and GalNP(rho) (curves c and d, respectively). For these materials, the reflections (110) and (200) are lost, most likely due to a reduction of contrast. Nevertheless, the presence of the d_{100} peak in the XRD patterns in all cases indicated that the process of pore loading and the additional functionalization with the corresponding saccharides, did not to a large extent modify the mesoporous scaffolding. GosNP(icg), GalNP(dox) and GalNP(nav) show XRD profiles similar to those from GosNP(rho) and GalNP(rho).

C, TEM analysis showing the presence of the mesoporous structure in the final functionalized solids. The typical channels of the MSNs matrix are visualized as alternate black and white stripes (NPs calcined (a) and GalNP(rho) (b)). The figure also shows that the prepared NPs-based materials are obtained as spherical particles with diameter ca. 100 nm. GosNP(rho), GosNP(icg), GalNP(dox) and GalNP(nav) show TEM images similar to this for GalNP(rho). Scale bars: 50 nm.

D, Panel a, N_2 adsorption-desorption isotherms of the calcined NPs. A typical curve for these mesoporous solids consisting of an adsorption step at intermediate P/P_0 value (0.1-0.3) can be observed. This curve corresponds to a type IV isotherm, in which the observed step deals with nitrogen condensation inside the mesopores. The absence of a hysteresis loop in this interval and the narrow BJH pore distribution suggest the existence of uniform cylindrical mesopores (pore diameter of 2.51 nm and pore volume of 0.72 cm^3 g^{-1} calculated by using the BJH model on the adsorption branch of the isotherm). The application of the BET model resulted in a value of 996 m^2/g for the total specific surface. Panels b and c, N_2 adsorption-desorption isotherm of GosNP(rho) and GalNP(rho), respectively. The isotherms are typical of mesoporous systems with practically filled mesopores. Consequently, relatively low N_2 adsorbed volume (BJH mesopore volume = 0.436 cm^3 g^{-1} and 0.418 cm^3 g^{-1} respectively) and surface area (197 m^2/g and 373 m^2/g) values were calculated. These solids show flat curves when compared (at the same scale) to those of the NPs parent material, which indicates significant pore blocking and the subsequent absence of significant mesoporosity. GosNP(icg), GalNP(dox) and GalNP(nav) show similar N_2 adsorption-desorption isotherms to that shown by GosNP(rho) and GalNP(rho).

E, Cargo release studies of the GosNP(rho), GosNP(icg), GalNP(rho), GalNP(dox) and GalNP(nav) beads by spectrofluorometry. The graphs represent the release profiles of

the cargoes in the absence (a) or the presence (b) of β−galactosidase from *Aspergillus oryzae* in water at pH 4.5 at room temperature at the indicated time points.

F, NCI-H226 lung squamous carcinoma cells were treated with palbociclib (1 μM) for 1 week, and senescence induction was assessed by SAβGal staining. Next, cultures were exposed to GalNP(rho) (50 μg/ml, for 16 h). Pictures show representative images illustrating rhodamine release by confocal microscopy. Cells were co-stained with Calcein and nuclei were stained with Hoechst. Rhodamine intensity relative to cell surface was quantified using Definiens software. Graphs to the left right show the percentage of senescent cells with rhodamine intensity > 95% of the non-senescent cells (control) cells. Each assay was repeated at least three times with similar results. Scale bar: 50 μm.

G, UT-SCC-42B head and neck squamous carcinoma cells were treated with palbociclib and GalNP(rho) as in **F**. The graph shows the percentage of senescent cells with rhodamine intensity > 95% of the non-senescent cells (control) cells. Each assay was repeated at least three times with similar results.

H, SK-MEL-103 cells were treated with palbociclib (5 μM) for 2 weeks and exposed to GalNP(rho) (1 mg/ml filtered, for 16 h). Lysosomes were stained with lysotracker green for colocalization studies. Scale bar: 50 μm

I, Subcutaneous SK-MEL-103 tumor xenograft, liver and spleen of athymic nude mice treated with palbociclib and GalNP(rho) as in **Figure 1D**. At 6 h post-injection, mice were sacrificed, tumors and organs were collected, and rhodamine fluorescence was analyzed by an IVIS spectrum imaging system.

J, Subcutaneous tumor xenografts of SK-MEL-103 melanoma cells in athymic nude mice. Upon tumor formation (average volume 200 mm^3), mice were treated daily with palbociclib (oral gavage, 80 mg/kg) during 7 days. Mice were tail vein injected with 150 μl of a solution containing GalNP(icg) (10 mg/ml). At 16 h post-injection, mice were sacrificed, tumors were collected, and fluorescence was analyzed by an IVIS spectrum imaging system.

K, Subcutaneous tumor xenografts of NCI-H226 lung squamous carcinoma cells in athymic nude mice. Upon tumor formation (average volume 200 mm^3), mice were treated daily with palbociclib (oral gavage, 100 mg/kg) during 15 days (weekdays). Mice were tail vein injected with 200 μl of a solution containing GalNP(icg) (10 mg/ml). At 16 h post-injection, mice were sacrificed, tumors were collected, and fluorescence was analyzed by an IVIS spectrum imaging system.

Appendix Figure S2
(page 1 out of 2)

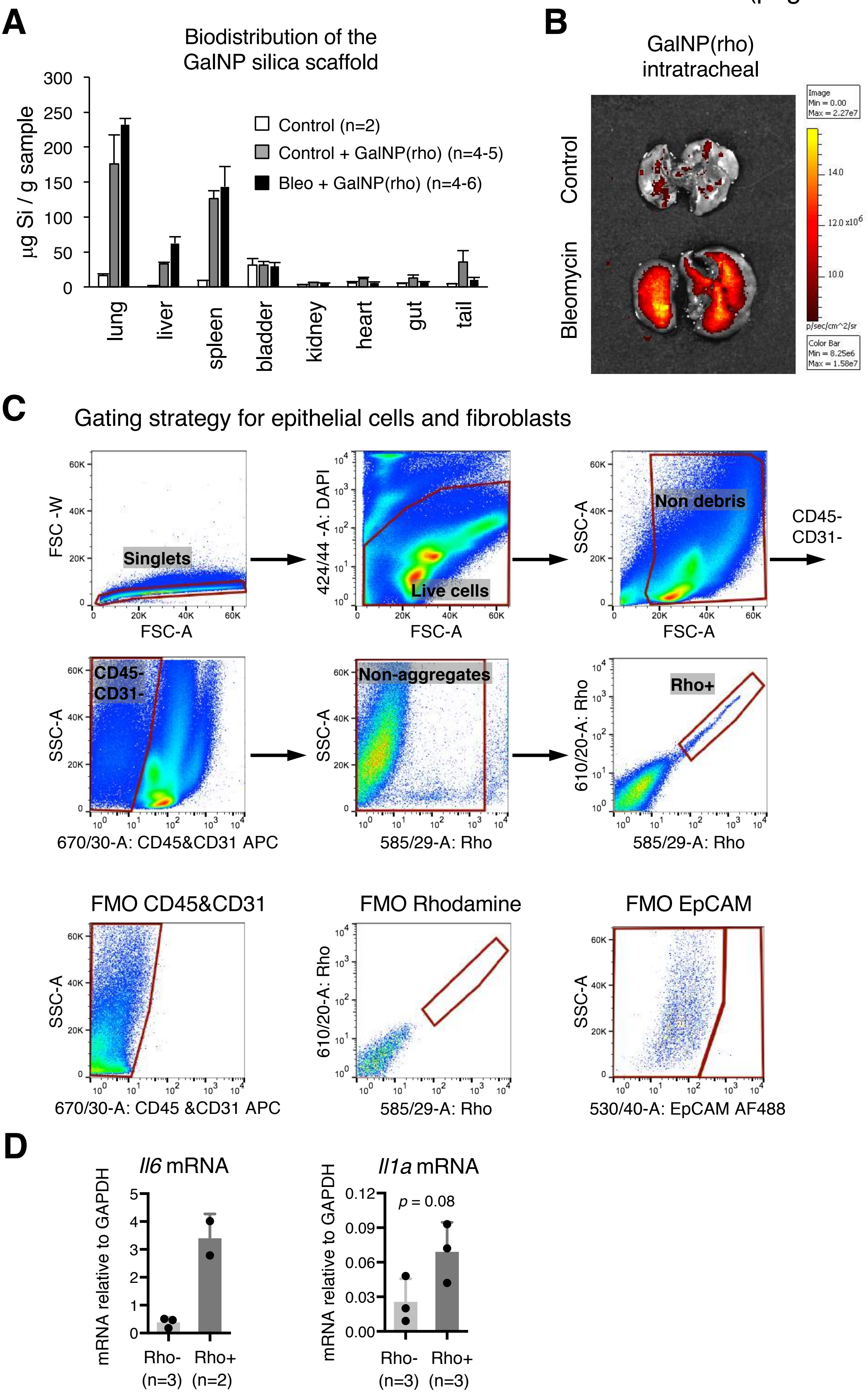

Appendix Figure S2
(page 2 out of 2)

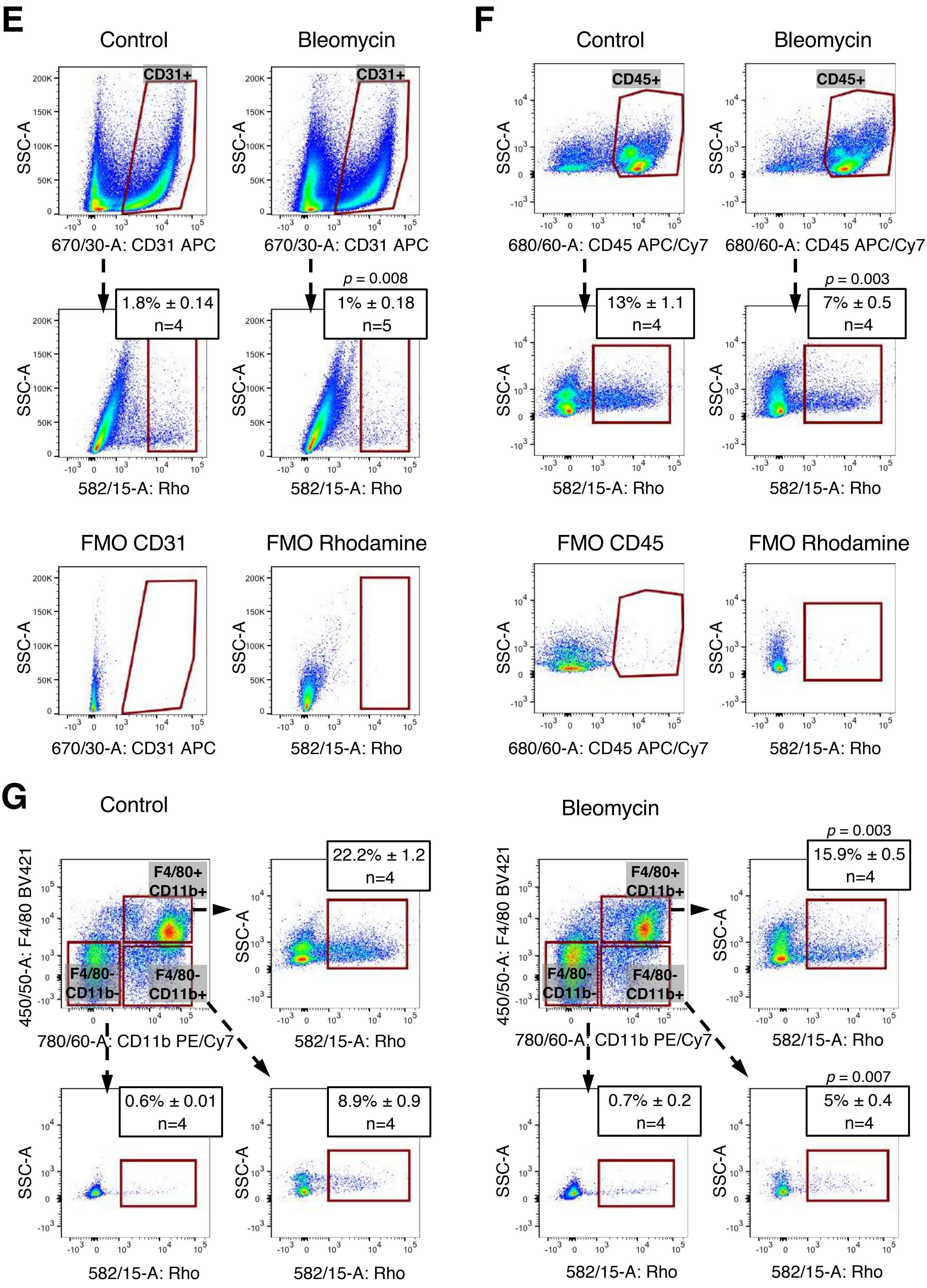

Appendix Figure S2. Release of gal-encapsulated fluorophores in fibrotic lungs

A, C57BL/6 mice were subjected to a single intratracheal administration of bleomycin (1.5 U/kg). 2 weeks later, mice were intratracheally injected with 30 µl of a solution containing GalNP(rho) (4 mg/ml). At 6 h post-inoculation, mice were sacrificed and the lungs were analyzed by an IVIS spectrum imaging system.

B, Biodistribution studies of the GalNP silica scaffold in lung, liver, spleen, bladder, kidney, heart, gut and tail. Control and bleomycin-treated C57BL/6 mice were tail vein injected with 200 µl of a solution containing GalNP(rho) (4 mg/ml). At 6 h post-GalNP(rho) injection the indicated organs were extracted for Si detection. The graph shows the µg Si per g of sample detected by Inductively Coupled Plasma Mass Spectroscopy (ICP-MS) in the indicated organs.

C, Gating strategy used to exclude endothelial and haematopoietic cells. The example is illustrated with the same bleomycin-treated lung shown in **Fig. 2D**. Cells were sorted using pulse processing to exclude cell aggregates; DAPI was used as dead discriminator; and CD45 and CD31 were used to exclude endothelial (CD31+) and haematopoietic cells (CD45+). Aggregates of GalNP beads were excluded from the analysis as indicated. The lower panels show the fluorescence minus-one (FMO) controls of the indicated fluorophores.

D, Levels of *Il6* and *Il1α* mRNA measured by quantitative PCR in the indicated lung cell populations (Rho+ or Rho-, all CD45-CD31-) from mice treated with bleomycin as in the above panels. *Gapdh* was used for input normalization. Values are expressed as mean ± SD, and statistical significance was assessed by the two-tailed Student's t test.

E, Lung cell suspensions from control and bleomycin-treated mice were analyzed by flow cytometry. The upper panels show representative dot plots of rhodamine staining endothelial (CD31+) cells. Values in boxes correspond to the mean ± SEM, and statistical significance was assessed by the two-tailed Student's t test.

F, Lung cell suspensions from control and bleomycin-treated mice were analyzed by flow cytometry. The upper panels show representative dot plots of rhodamine staining haematopoietic (CD45+) cells. Values in boxes correspond to the mean ± SEM, and statistical significance was assessed by the two-tailed Student's t test: $^{**}p < 0.01$.

G, The upper panels show representative dot plots of F4/80 and CD11b staining of the CD45+ cells of panel **F**. Panels show rhodamine staining in macrophages (CD45+F4/80+CD11b+), granulocytes (CD45+F4/80-CD11b+) and lymphocytes (CD45+F4/80-CD11b-). Values in boxes correspond to the mean ± SEM, and statistical significance was assessed by the two-tailed Student's t test.

Appendix Figure S3
(page 1 out of 3)

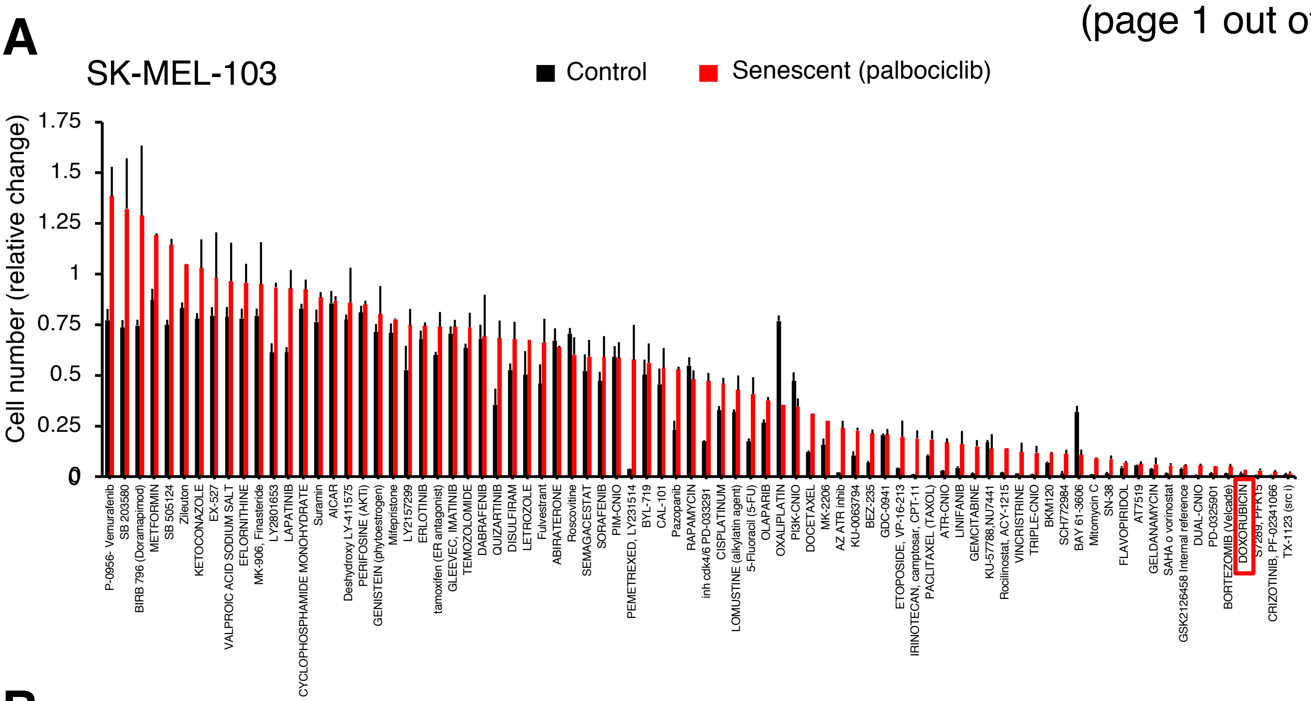

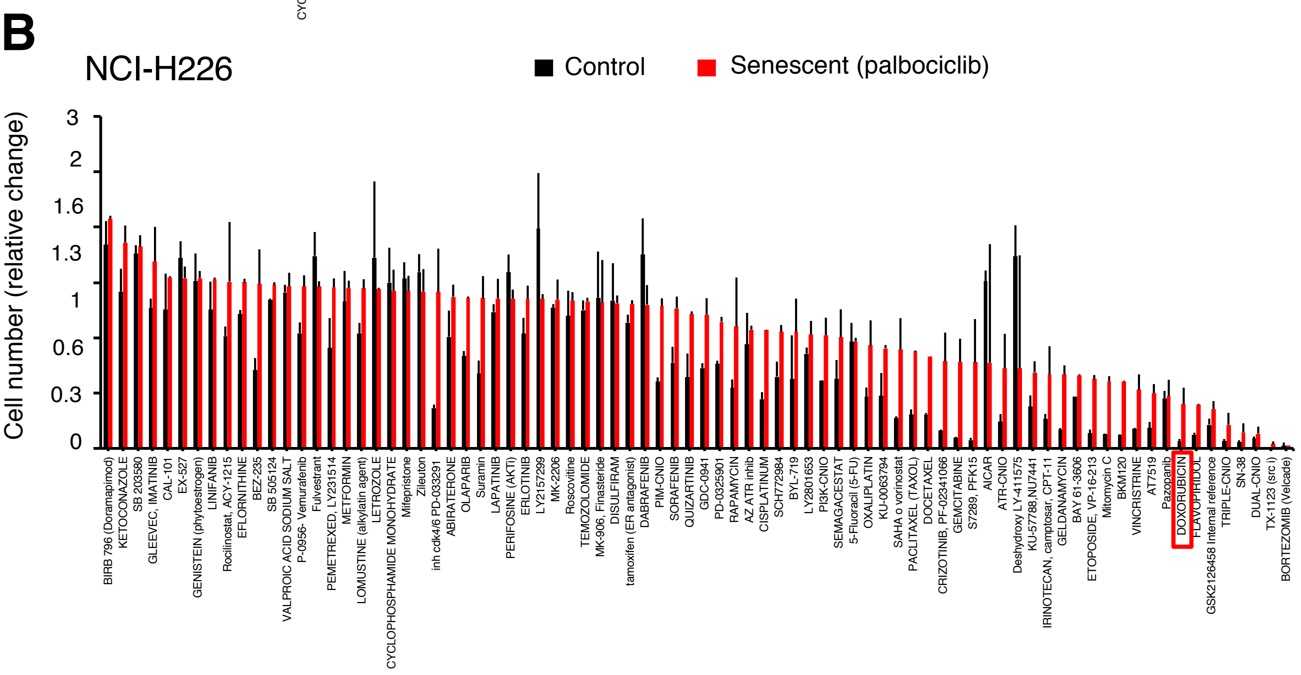

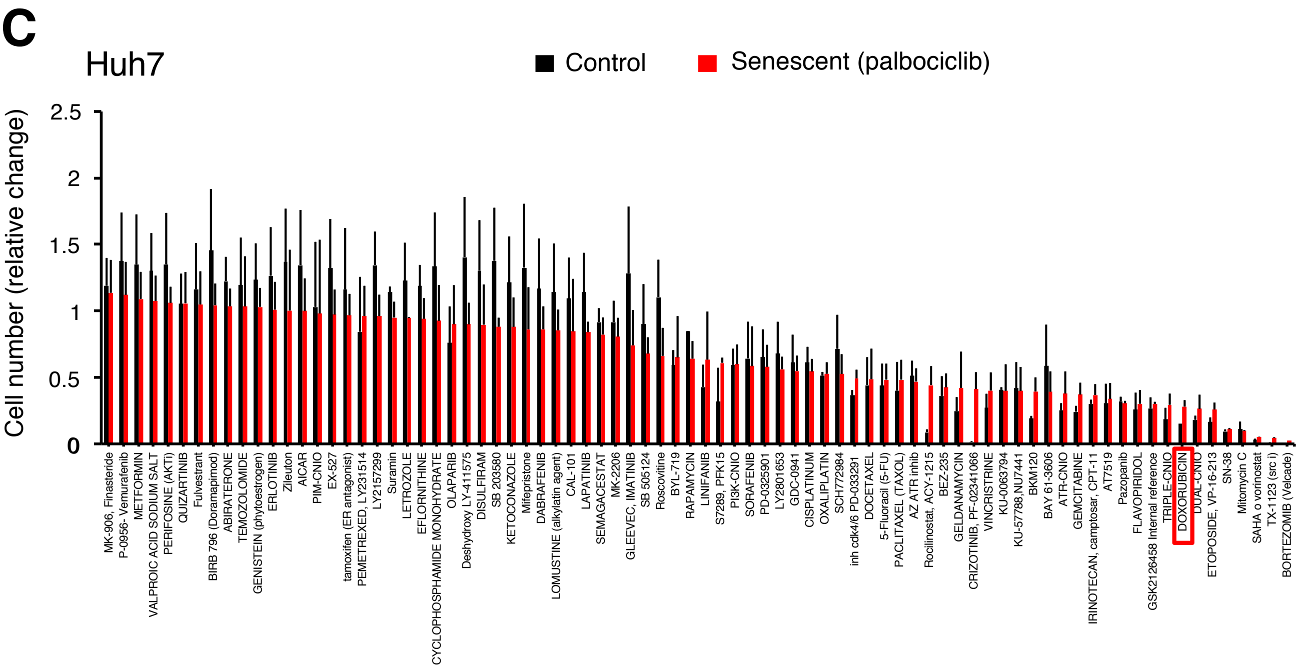

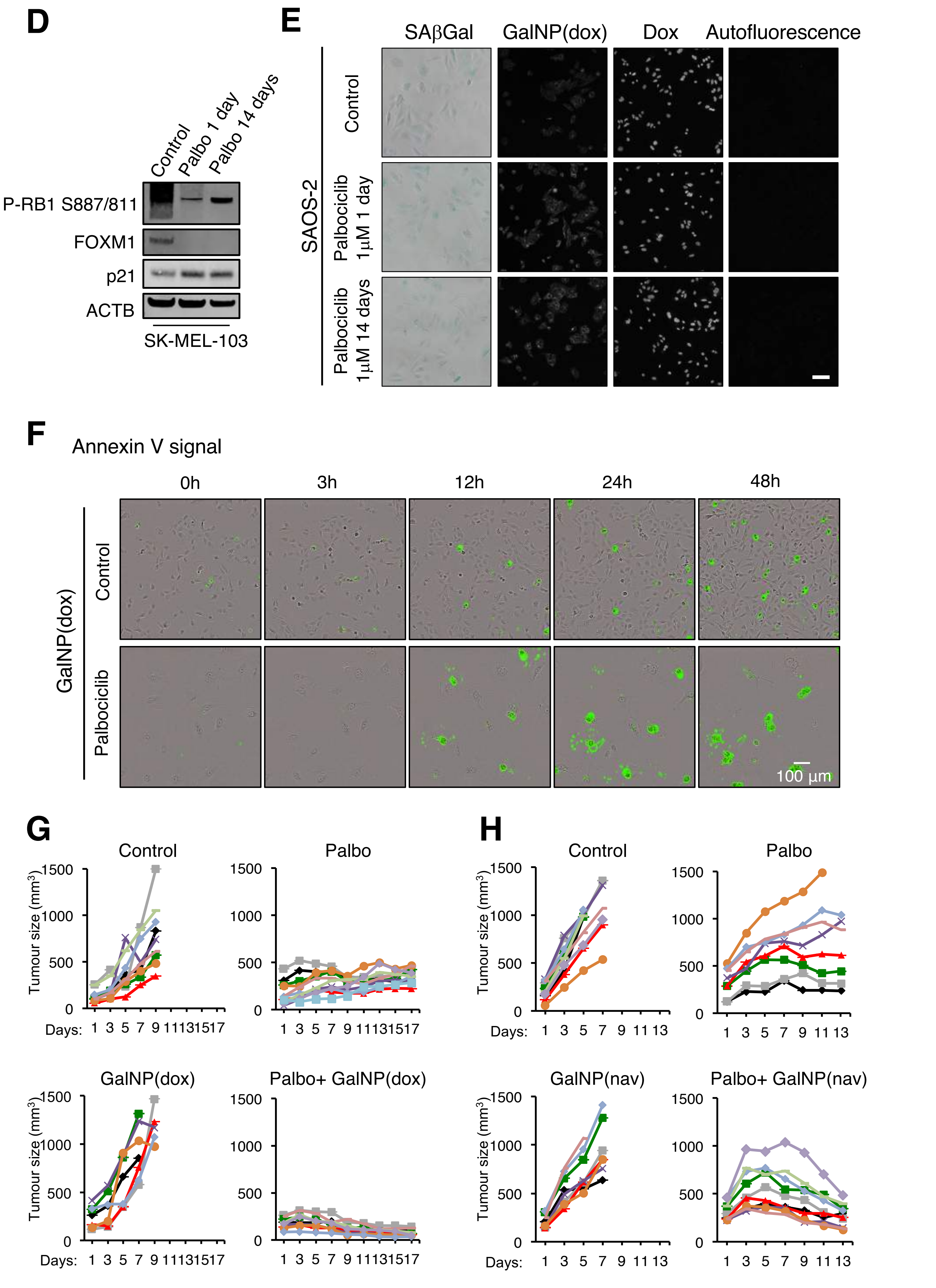
D
Control
Palbo 1 day
Palbo 14 days
P-RB1 S887/811
FOXM1
p21
ACTB
SK-MEL-103
E
SAβGal
GalNP(dox)
Dox
Autofluorescence
SAOS-2
Control
Palbociclib 1μM 1 day
Palbociclib 1μM 14 days
F
Annexin V signal
0h
3h
12h
24h
48h
GalNP(dox)
Control
Palbociclib
100 μm
G
Control
Palbo
GalNP(dox)
Palbo+ GalNP(dox)
Tumour size (mm³)
1500
1000
500
0
Days: 1 3 5 7 9 11 13 15 17
H
Control
Palbo
GalNP(nav)
Palbo+ GalNP(nav)
Days: 1 3 5 7 9 11 13

I

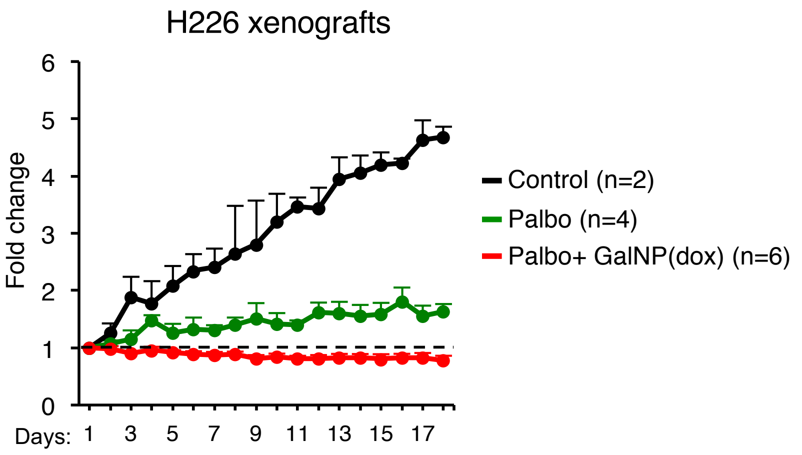
H226 xenografts
Fold change
6
5
4
3
2
1
0
Days: 1 3 5 7 9 11 13 15 17
Control (n=2)
Palbo (n=4)
Palbo+ GalNP(dox) (n=6)

J

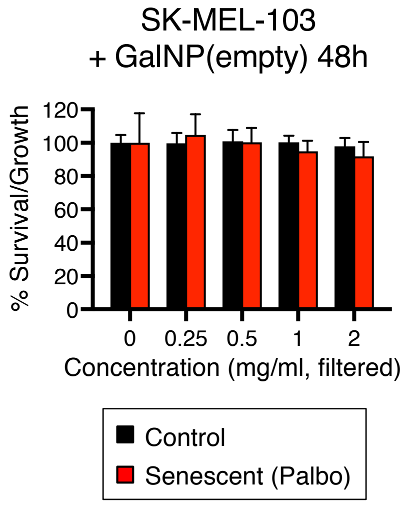
SK-MEL-103
+ GalNP(empty) 48h
% Survival/Growth
120
100
80
60
40
20
0
0 0.25 0.5 1 2
Concentration (mg/ml, filtered)
Control
Senescent (Palbo)

K

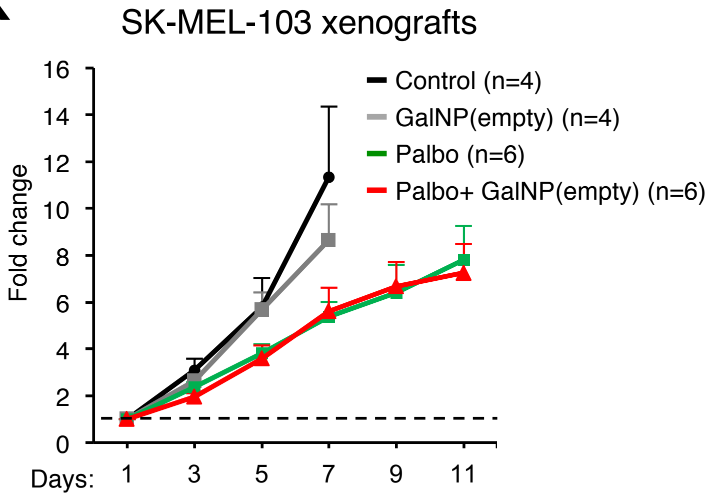
SK-MEL-103 xenografts
Fold change
16
14
12
10
8
6
4
2
0
Days: 1 3 5 7 9 11
Control (n=4)
GalNP(empty) (n=4)
Palbo (n=6)
Palbo+ GalNP(empty) (n=6)

Appendix Figure S3. Therapeutic activity of gal-encapsulated cytotoxic drugs in xenografts

A, Drug screening assay in control and senescent SK-MEL-103 cells. The graph shows the cell number (fold change) normalized to the control of untreated cells (DMSO only) with the indicated drugs.

B, Drug screening assay in control and senescent NCI-H226 cells, as in **A**.

C, Drug screening assay in control and senescent Huh7 cells, as in **A**.

D, Western blot analysis in SK-MEL-103 cells for P-Rb, FOXM1, p21 and ACTB upon the indicated treatments.

E, SAOS-2 osteosarcoma cells were treated with palbociclib (1 μM) for 1 or 14 days, and subjected to SAβgal staining. Next, cultures were exposed to free doxorubicin 50 (μM) or GalNP(dox) (1 mg/ml, filtered) for 30 min. Pictures show representative images by confocal microscopy. Scale bar: 50 μm.

F, Annexin V signal of SK-MEL-103 cells of **Figure 3B** at the indicated time points, analyzed by confocal microscopy.

G, Individual tumor size measurements of **Figure 3C** with the indicated treatments.

H, Individual tumor size measurements of **Figure 3D** with the indicated treatments.

I, Mice carrying subcutaneous NCI-H226 xenografts (~200 mm^3 average volume at the start of treatments), were treated daily with palbociclib (oral gavage, 50 mg/kg) and/or GalNP(dox) (tail vein injection, 200 μl of a solution with 4 mg/ml of GalNP containing a total of 1 mg/kg of deliverable doxorubicin), alone or in combination, as indicated, for 19 days. For each tumor, the relative tumor volume change was calculated relative to its baseline prior to treatment. Values are expressed as mean ± SEM.

J, SK-MEL-103 cells were treated with palbociclib (1 μM) for 14 days, and plated in 96 well plates. GalNP(empty) were diluted in DMEM at the indicated concentrations, and added to the cultures after filtration though a 0.45 μm filter. Viability was assessed 48 hours later with a CellTiter-GLO Luminescent Cell Viability Assay.

K, Mice carrying subcutaneous SK-MEL-103 xenografts (~150 mm^3 average volume at the start of treatments), were treated daily with palbociclib (oral gavage, 50 mg/kg) and/or GalNP(empty) (tail vein injection, 200 μl of a solution with 4 mg/ml of GalNP), alone or in combination, as indicated, for 11 days. For each tumor, the relative tumor volume change was calculated relative to its baseline prior to treatment. Values are expressed as mean ± SEM.

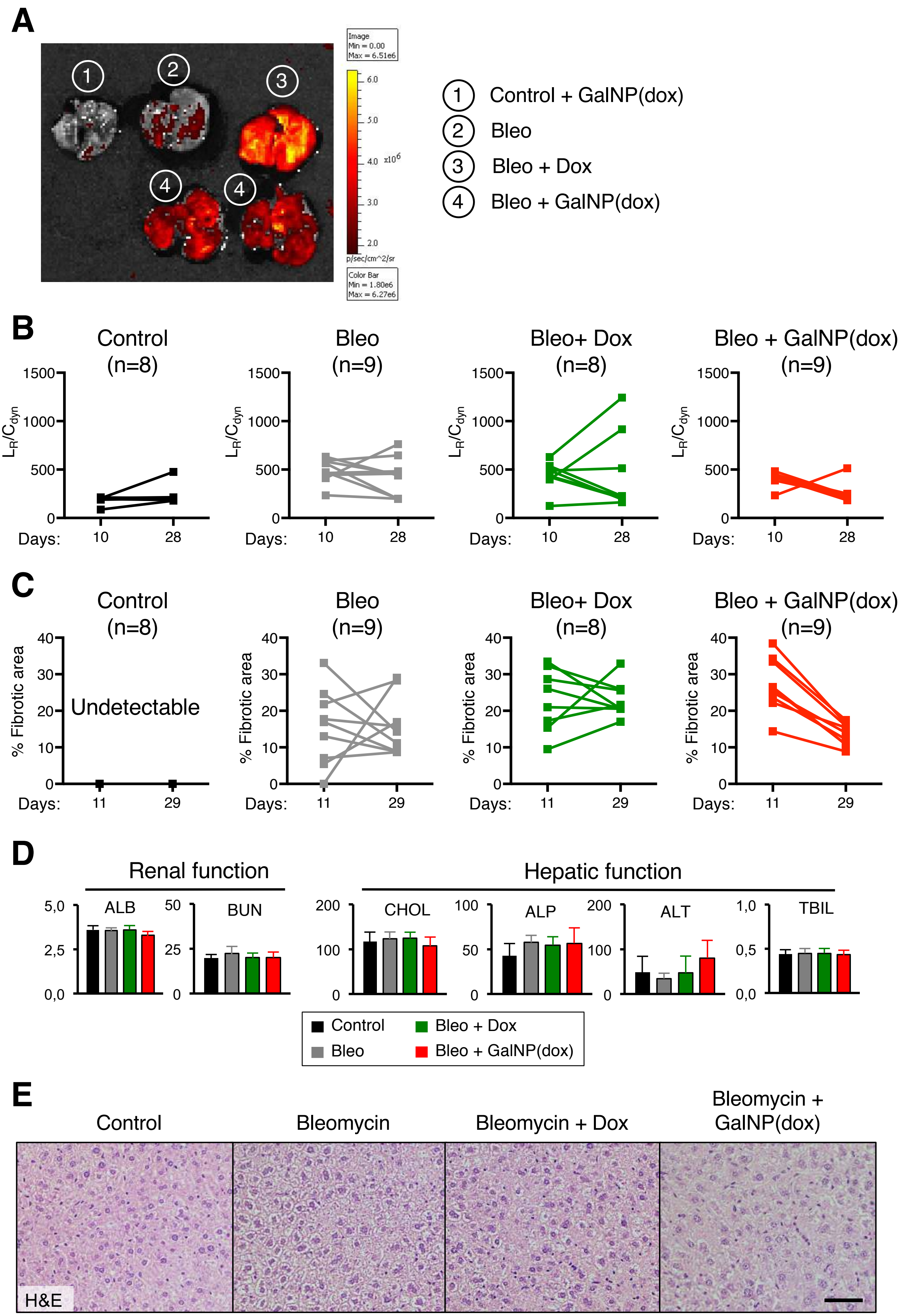
A
1
2
3
4
4
Image
Min = 0.00
Max = 6.51e6
6.0
5.0
4.0 x10^6
3.0
2.0
p/sec/cm^2/sr
Color Bar
Min = 1.80e6
Max = 6.27e6
1 Control + GalNP(dox)
2 Bleo
3 Bleo + Dox
4 Bleo + GalNP(dox)
B
Control (n=8)
Bleo (n=9)
Bleo+ Dox (n=8)
Bleo + GalNP(dox) (n=9)
L_R/C_dyn
1500
1000
500
0
Days: 10 28
C
Control (n=8)
Bleo (n=9)
Bleo+ Dox (n=8)
Bleo + GalNP(dox) (n=9)
% Fibrotic area
40
30
20
10
0
Undetectable
Days: 11 29
D
Renal function
Hepatic function
ALB
BUN
CHOL
ALP
ALT
TBIL
5,0
2,5
0,0
50
25
0
200
100
0
100
50
0
200
100
0
1,0
0,5
0,0
Control
Bleo
Bleo + Dox
Bleo + GalNP(dox)
E
Control
Bleomycin
Bleomycin + Dox
Bleomycin + GalNP(dox)
H&E

Appendix Figure S4. Therapeutic activity of gal-encapsulated doxorubicin on pulmonary fibrosis

A, C57BL/6 male mice were subjected to a single intratracheal administration of bleomycin at 1.5 U/kg BW. Beginning at day 14, mice were treated every four days with free doxorubicin (tail vein injection, 4 mg/kg) or every two days with GalNP(dox) (tail vein injection, 200 μl of a solution with 4 mg/ml of GalNP containing a total of 1 mg/kg of deliverable doxorubicin) for 14 days, that is, until day 28 post-bleomycin. At the end of the treatment mice were sacrificed, lungs were collected, and doxorubicin fluorescence was analyzed by an IVIS spectrum imaging system.

B, Individual L_R/C_{dyn} values of **Figure 4B** with the indicated treatments.

C, Individual values of % fibrotic area of **Figure 4C** with the indicated treatments.

D, Analysis of renal and hepatic function of mice as in **Figure 4** at the end of the treatment. ALB, albumin; BUN, blood urea nitrogen; CHOL, cholesterol; ALP, alkaline phosphatase; ALT, alanine transaminase; TBIL, total bilirubin.

E, Haematoxylin and eosin (HE) staining of the livers of mice as in **Figure 4** at the end of the treatment. Scale bar: 500 μm.

Appendix Table S1. BET specific surface values, pore volumes and pore sizes calculated from the N_2 adsorption-desorption isotherms for selected materials.

	S_{BET} (m^2 g^{-1})	Pore Volume [a] (m^2 g^{-1})	Pore size [a,b] (nm)
**NPs**	996	0.720	2.51
**GosNP(rho)**	197	0.436	---
**GalNP(rho)**	372	0.418	---

[a] Pore volumes and pore sizes are only associated with intraparticle mesopores. [b]Pore size estimated by using the BJH model applied on the adsorption branch of the isotherm.

Appendix Table S2. Content (α) in mg of 'saccharide' and cargo for solids **GosNP(rho)**, **GosNP(icg)**, **GalNP(rho)**, **GalNP(dox)** and **GalNP(nav)**.

Solid	$\alpha_{\text{'saccharide'}}$ (mg/g solid)	α_{cargo} (mg/g solid)
**GosNP(rho)**	141.7	97.80
**GosNP(icg)**	151.4	43.30
**GalNP(rho)**	137.8	147.9
**GalNP(dox)**	192.1	101.3
**GalNP(nav)**	183.6	97.20

Appendix Table S3. Amount (in mg (α) or as percentage (%)) of maximum drug release for solids GalNP(dox) and GalNP(nav).

Solid	α_{drug} (mg/g solid)	% of loaded drug that is released
**GalNP(dox)**	31.05	30.65
**GalNP(nav)**	33.56	34.53
