# Supplementary figures and images for: A versatile drug delivery system targeting senescent cells
Source: EMBO Mol Med. 2018 Jul 16;10(9):e9355. doi: 10.15252/emmm.201809355 (PMC6127887; doi:10.15252/emmm.201809355)

High brightness low contrast:

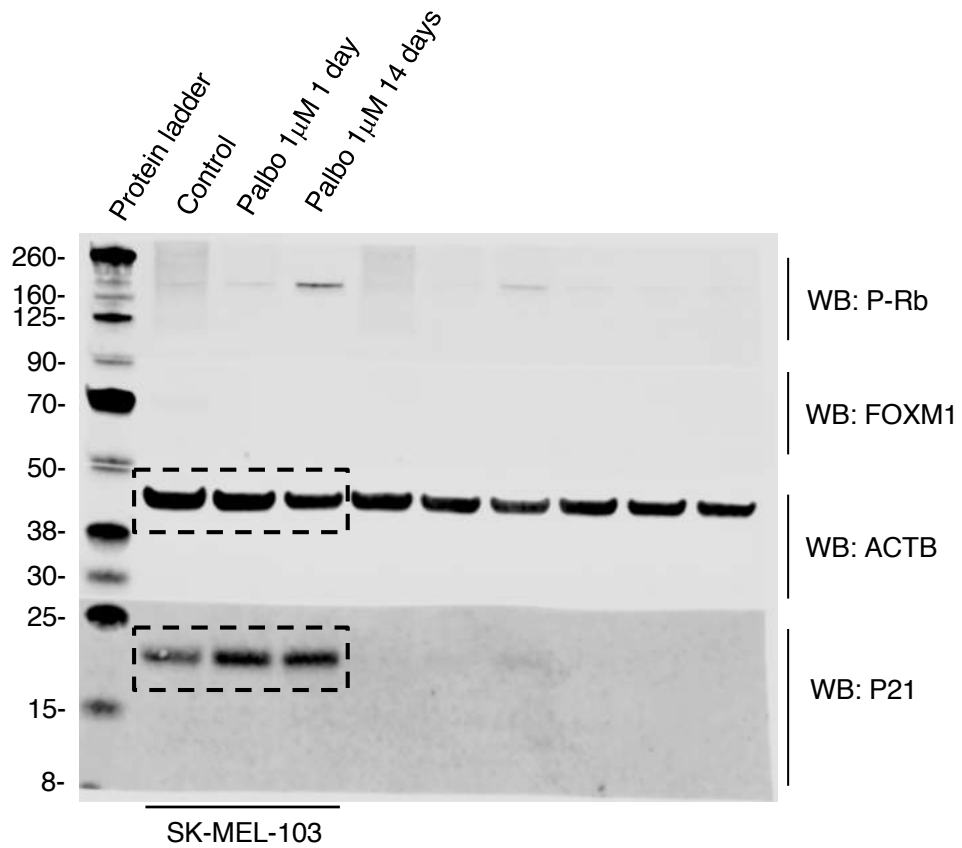

Low brightness high contrast:

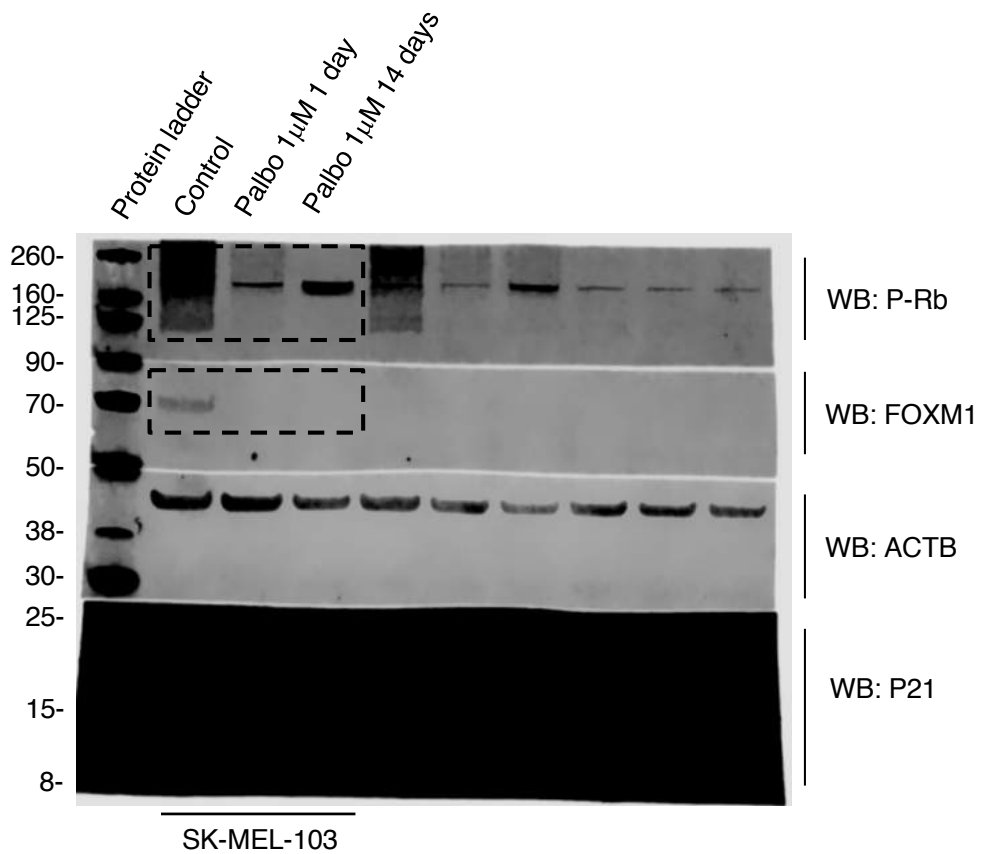

Supplement: Supplementary file 4 — Source Data for Appendix [file EMMM-10-e9355-s003.pdf]
