# Supplementary material for: A versatile drug delivery system targeting senescent cells
Source: EMBO Mol Med. 2018 Jul 16;10(9):e9355. doi: 10.15252/emmm.201809355 (PMC6127887; doi:10.15252/emmm.201809355)
Supplement: Supplementary file 3 — Review Process File [file EMMM-10-e9355-s004.pdf]

EMBO Molecular Medicine

A versatile drug delivery system targeting senescent cells

Daniel Muñoz-Espín, Miguel Rovira, Irene Galiana, Cristina Giménez, Beatriz Lozano-Torres, Marta Paez-Ribes, Susana Llanos, Selim Chaib, Maribel Muñoz-Martín, Alvaro C. Ucero, Guillermo Garaulet, Francisca Mulero, Stephen Dann, Todd VanArsdale, David J. Shields, Andrea Bernardos, José Ramón Murguía, Ramón Martínez-Máñez, Manuel Serrano

Review timeline:

Submission date:	11 June 2018
Editorial Decision:	14 June 2018
Revision received:	20 June 2018
Accepted:	22 June 2018

Editor: Céline Carret

Transaction Report:

Note: Please note that the manuscript was previously reviewed at another journal and the reports were taken into account in the decision making process at EMBO Molecular Medicine. Since the original reviews are not subject to EMBO's transparent review process policy, the reports and author response cannot be published.

EMBO PRESS

YOU MUST COMPLETE ALL CELLS WITH A PINK BACKGROUND ↓

PLEASE NOTE THAT THIS CHECKLIST WILL BE PUBLISHED ALONGSIDE YOUR PAPER

Corresponding Author Name: Manuel Serrano and Daniel Muñoz-Espín

Journal Submitted to: EMBO Molecular Medicine

Manuscript Number: EMM-2018-09355

Reporting Checklist For Life Sciences Articles (Rev. June 2017)

This checklist is used to ensure good reporting standards and to improve the reproducibility of published results. These guidelines are consistent with the Principles and Guidelines for Reporting Preclinical Research issued by the NIH in 2014. Please follow the journal's authorship guidelines in preparing your manuscript.

A- Figures

1. Data

The data shown in figures should satisfy the following conditions:

- the data were obtained and processed according to the field's best practice and are presented to reflect the results of the experiments in an accurate and unbiased manner.
- figure panels include only data points, measurements or observations that can be compared to each other in a scientifically meaningful way.
- graphs include clearly labeled error bars for independent experiments and sample sizes. Unless justified, error bars should not be shown for technical replicates.
- if n< 5, the individual data points from each experiment should be plotted and any statistical test employed should be justified
- Source Data should be included to report the data underlying graphs. Please follow the guidelines set out in the author ship guidelines on Data Presentation.

2. Captions

Each figure caption should contain the following information, for each panel where they are relevant:

- a specification of the experimental system investigated (eg cell line, species name).
- the assay(s) and method(s) used to carry out the reported observations and measurements
- an explicit mention of the biological and chemical entity(ies) that are being measured.
- an explicit mention of the biological and chemical entity(ies) that are altered/varied/perturbed in a controlled manner.

- the exact sample size (n) for each experimental group/condition, given as a number, not a range;
- a description of the sample collection allowing the reader to understand whether the samples represent technical or biological replicates (including how many animals, litters, cultures, etc.).
- a statement of how many times the experiment shown was independently replicated in the laboratory.
- definitions of statistical methods and measures:
  - common tests, such as t-test (please specify whether paired vs. unpaired), simple χ2 tests, Wilcoxon and Mann-Whitney tests, can be unambiguously identified by name only, but more complex techniques should be described in the methods section;
  - are tests one-sided or two-sided?
  - are there adjustments for multiple comparisons?
  - exact statistical test results, e.g., P values = x but not P values < x;
  - definition of 'center values' as median or average;
  - definition of error bars as s.d. or s.e.m.

Any descriptions too long for the figure legend should be included in the methods section and/or with the source data.

USEFUL LINKS FOR COMPLETING THIS FORM

http://www.antibodypedia.com
http://1degreebio.org
http://www.equator-network.org/reporting-guidelines/improving-bioscience-research-repo
http://grants.nih.gov/grants/olaw/olaw.htm
http://www.mrc.ac.uk/Ourresearch/Ethicsresearchguidance/Useofanimals/index.htm
http://ClinicalTrials.gov
http://www.consort-statement.org
http://www.consort-statement.org/checklists/view/32-consort/66-title
http://www.equator-network.org/reporting-guidelines/reporting-recommendations-for-tum
http://datadryad.org
http://figshare.com
http://www.ncbi.nlm.nih.gov/gap
http://www.ebi.ac.uk/ega
http://biomodels.net/
http://biomodels.net/miriam/
http://jjj.biochem.sun.ac.za
http://oba.od.nih.gov/biosecurity/biosecurity_documents.html
http://www.selectagents.gov/

In the pink boxes below, please ensure that the answers to the following questions are reported in the manuscript itself. Every question should be answered. If the question is not relevant to your research, please write NA (non applicable). We encourage you to include a specific subsection in the methods section for statistics, reagents, animal models and human subjects.

B- Statistics and general methods

Please fill out these boxes ↓ (Do not worry if you cannot see all your text once you press return)

1.a. How was the sample size chosen to ensure adequate power to detect a pre-specified effect size?	In cell culture experiments, at least three independent replicates were assayed to ensure reproducibility. In animal experiments, sample sizes for comparisons between treatments followed Mead's recommendations (Festing et al., 2002).
1.b. For animal studies, include a statement about sample size estimate even if no statistical methods were used.	In animal studies, the accumulated n value (N) for a given comparison minus the number of groups or treatments (T) was between 10 and 20 in most of the experiments, as recommended. In the case of bleomycin-induced pulmonary fibrosis experiments, the n value was higher due to the intrinsically high variability of the assay.
2. Describe inclusion/exclusion criteria if samples or animals were excluded from the analysis. Were the criteria pre-established?	For in vivo studies, mice were excluded if they developed humane-intervention end-point complications in the course of the study. These criteria were pre-established.
3. Were any steps taken to minimize the effects of subjective bias when allocating animals/samples to treatment (e.g. randomization procedure)? If yes, please describe.	For cell culture studies, the treatment order of the wells were pre-determined. For in vivo studies, all mice were age and weight matched.
For animal studies, include a statement about randomization even if no randomization was used.	All mice were age and weight matched. No additional randomization was used for animal studies.
4.a. Were any steps taken to minimize the effects of subjective bias during group allocation or/and when assessing results (e.g. blinding of the investigator)? If yes please describe.	For cell culture fluorescence analysis, quantifications wered performed in a blinded manner. For plethysmography measurements and CT analysis, the evaluators were blind to the treatments. For the xenograft assays, investigators were not blinded to the experimental groups.
4.b. For animal studies, include a statement about blinding even if no blinding was done	In the animal studies, blinding was performed as follows: for plethysmography measurements and CT analysis, the evaluators were blind to the treatments; for the xenograft assays, investigators were not blinded to the experimental groups.
5. For every figure, are statistical tests justified as appropriate?	Statistical tests were performed only on experiments with at least three replicates. All the statistical tests used (Student's t-tests, Fisher exact test, and one-way ANOVA) and are justified as appropiate.
Do the data meet the assumptions of the tests (e.g., normal distribution)? Describe any methods used to assess it.	Normal distribution was tested using the Shapiro-Wilk test, and was confirmed in the majority of data.
Is there an estimate of variation within each group of data?	Equal variance was tested using the F-test, and was confirmed in the majority of data sets.
Is the variance similar between the groups that are being statistically compared?	The variance between the groups was similar in the majority of data.

C- Reagents

6. To show that antibodies were profiled for use in the system under study (assay and species), provide a citation, catalog number and/or clone number, supplementary information or reference to an antibody validation profile. e.g., Antibodypedia (see link list at top right), 1DegreeBio (see link list at top right).	For immunohistochemistry on paraffin sections, the following antibodies were used: Ki67 (Master Diagnostica, #0003110QD), phosphorylated Rb (Ser807/811) (Cell Signalling Technology, #9308). For flow cytometry, the following rat monoclonal conjugated antibodies were used: antiCD31 APC (1:400, BD Biosciences; #551260), antiCD45 APC (1:200, BD Biosciences, #559864), antiCD326 (EpCAM) AF488 (1:200, Biolegend, #118210), antiCD45.2 APC-Cy7 (1:100, Biolegend, #109824), antiF4/80 BV421 (1:50, Biolegend, 123131), antiCD11b PE-Cy7 (1:200, BD Biosciences #552850). For western blot, the following primary antibodies were used: rabbit anti-P-Rb S887/811 (1:1000, Cell Signaling, #8516), rabbit anti-FoxM1 D1205 (1:5000, Cell Signaling, #5436), rabbit anti-ACTB GTU-88 (1:10000, Sigma, #T6557) and goat anti-p21 C-19-G (1:200, Santa Cruz, #sc-397-G). These antobodies were subsequently incubated with the corresponding secondary anti-IgG antibodies: anti-rabbit IRDye 680 CW (1:15000, LI-COR, #926-68071), anti-rabbit IRDye 800 CW (1:15000, LI-COR, #926-32211) and anti-goat IRDye 680 CW (1:10000, LI-COR, #926-68074).
7. Identify the source of cell lines and report if they were recently authenticated (e.g., by STR profiling) and tested for mycoplasma contamination.	Cell lines SK-MEL-103 (human melanoma), NCI-H226 (human lung squamous cell carcinoma), Huh7 (human hepatocarcinoma), and SAOS-2 (human primary osteogenic sarcoma) were obtained from ATCC. UT-SCC-42B cells (human head and neck squamous cell carcinoma) were kindly provided by Dr. Reidar Grenman (Turku University, Finland). Cells were routinely tested for mycoplasma contamination using the mycoplasma tissue culture NI (MTC-NI) Rapid Detection System (Gen-Probe).

* for all hyperlinks, please see the table at the top right of the document

D- Animal Models

8. Report species, strain, gender, age of animals and genetic modification status where applicable. Please detail housing and husbandry conditions and the source of animals.	All mice were maintained at the Spanish National Cancer Research Centre (CNIO) under specific pathogen-free conditions in accordance with the recommendations of the Federation of European Laboratory Animal Science Associations (FELASA). Mice were housed in ventilated racks with integration of Individually Ventilated Caging (IVC) units in the building ventilation systems. 8- to 10-week-old athymic nude female mice (Hsd:Athymic Nude-Foxn1nu) were purchased from Envigo. 8- to 10-week-old male C57BL/6 wild-type mice were in-house bred at the CNIO.
9. For experiments involving live vertebrates, include a statement of compliance with ethical regulations and identify the committee(s) approving the experiments.	All animal experiments were approved by the Ethical Committee for Research and Animal Welfare (CEIyBA) and performed in accordance with the guidelines stated in the International Guiding Principles for Biomedical Research Involving Animals, developed by the Council for International Organizations of Medical Sciences (CIOMS).
10. We recommend consulting the ARRIVE guidelines (see link list at top right) (PLoS Biol. 8(6), e1000412, 2010) to ensure that other relevant aspects of animal studies are adequately reported. See author guidelines, under 'Reporting Guidelines'. See also: NIH (see link list at top right) and MRC (see link list at top right) recommendations. Please confirm compliance.	All studies were performed in compliance with the ARIIVE guidelines and the NIH and MRC recommendations.

E- Human Subjects

11. Identify the committee(s) approving the study protocol.	N/A
12. Include a statement confirming that informed consent was obtained from all subjects and that the experiments conformed to the principles set out in the WMA Declaration of Helsinki and the Department of Health and Human Services Belmont Report.	N/A
13. For publication of patient photos, include a statement confirming that consent to publish was obtained.	N/A
14. Report any restrictions on the availability (and/or on the use) of human data or samples.	N/A
15. Report the clinical trial registration number (at ClinicalTrials.gov or equivalent), where applicable.	N/A
16. For phase II and III randomized controlled trials, please refer to the CONSORT flow diagram (see link list at top right) and submit the CONSORT checklist (see link list at top right) with your submission. See author guidelines, under 'Reporting Guidelines'. Please confirm you have submitted this list.	N/A
17. For tumor marker prognostic studies, we recommend that you follow the REMARK reporting guidelines (see link list at top right). See author guidelines, under 'Reporting Guidelines'. Please confirm you have followed these guidelines.	N/A

F- Data Accessibility

18: Provide a "Data Availability" section at the end of the Materials & Methods, listing the accession codes for data generated in this study and deposited in a public database (e.g. RNA-Seq data: Gene Expression Omnibus GSE39462, Proteomics data: PRIDE PXD000208 etc.) Please refer to our author guidelines for 'Data Deposition'.<br><br>Data deposition in a public repository is mandatory for:<br>a. Protein, DNA and RNA sequences<br>b. Macromolecular structures<br>c. Crystallographic data for small molecules<br>d. Functional genomics data<br>e. Proteomics and molecular interactions	The primary RNAseq data from this publication have been deposited to the GEO Omnibus database [https://www.ncbi.nlm.nih.gov/geo] and assigned the identifier GSE97685.
19. Deposition is strongly recommended for any datasets that are central and integral to the study; please consider the journal's data policy. If no structured public repository exists for a given data type, we encourage the provision of datasets in the manuscript as a Supplementary Document (see author guidelines under 'Expanded View' or in unstructured repositories such as Dryad (see link list at top right) or Figshare (see link list at top right).	Data have been deposited as indicated.
20. Access to human clinical and genomic datasets should be provided with as few restrictions as possible while respecting ethical obligations to the patients and relevant medical and legal issues. If practically possible and compatible with the individual consent agreement used in the study, such data should be deposited in one of the major public access-controlled repositories such as dbGAP (see link list at top right) or EGA (see link list at top right).	N/A
21. Computational models that are central and integral to a study should be shared without restrictions and provided in a machine-readable form. The relevant accession numbers or links should be provided. When possible, standardized format (SBML, CellML) should be used instead of scripts (e.g. MATLAB). Authors are strongly encouraged to follow the MIRIAM guidelines (see link list at top right) and deposit their model in a public database such as Biomodels (see link list at top right) or JWS Online (see link list at top right). If computer source code is provided with the paper, it should be deposited in a public repository or included in supplementary information.	N/A

G- Dual use research of concern

22. Could your study fall under dual use research restrictions? Please check biosecurity documents (see link list at top right) and list of select agents and toxins (APHIS/CDC) (see link list at top right). According to our biosecurity guidelines, provide a statement only if it could.	N/A
